# The safety, efficacy and cost-effectiveness of the Maxm Skate, a lower limb rehabilitation device for use following total knee arthroplasty: study protocol for a randomised controlled trial

**DOI:** 10.1186/s13063-018-3102-9

**Published:** 2019-01-10

**Authors:** Matthew G. Liptak, Annika Theodoulou, Billingsley Kaambwa, Steve Saunders, Scott W. Hinrichs, Richard J. Woodman, Jeganath Krishnan

**Affiliations:** 1 Orthopaedics SA, Adelaide, South Australia Australia; 2 The International Musculoskeletal Research Institute Inc., Adelaide, South Australia Australia; 3 College of Medicine and Public Health, Flinders University, Adelaide, Australia; 4 NIHR Oxford Biomedical Research Centre, Oxford University Hospitals NHS Foundation Trust, Oxford, UK; 5 Health Economics Unit, College of Medicine and Public Health, Flinders University, Adelaide, Australia; 6 Saunders Sports and Spinal, Adelaide, Australia; 7 Flinders Private Hospital, Adelaide, South Australia Australia; 8 Flinders Centre for Epidemiology and Biostatistics, College of Medicine, Flinders University, Adelaide, Australia

**Keywords:** Total knee arthroplasty, Rehabilitation, Home-based, Range of motion, Cost-effectiveness

## Abstract

**Background:**

Physical rehabilitation is required to enhance functional outcomes and overall recovery following total knee arthroplasty (TKA). However, there are no universally accepted clinical guidelines available to consistently structure rehabilitation for TKA patients. A common method is rehabilitation provided in an outpatient setting, on a one-to-one treatment basis. This method is resource-intensive and outcomes must be compared to less costly alternatives such as home-based rehabilitation. The current study will analyse a novel home-based rehabilitation program. The Maxm skate is a portable, lower-limb, postoperative, rehabilitation exercise device for individual use in a hospital or home-based setting. This study was developed to compare the safety, efficacy and cost-effectiveness of the Maxm Skate rehabilitation program to standard rehabilitative care following TKA. The primary outcome is the range of motion (ROM) achieved by patients who received the Maxm Skate program compared to standard care at three months post TKA. Secondary outcomes include patient-reported outcomes, costs and functional evaluations which will be collected at multiple time-points up to 12 months after TKA.

**Methods:**

This is a single-blinded, randomised controlled trial (RCT) in which 116 eligible participants consented for primary TKA will be randomly allocated to receive either the Maxm Skate rehabilitation program or standard rehabilitative care. Fifty-eight participants per group will provide 90% power (α = 0.05) to detect 10° of difference in ROM between groups at three months after TKA, assuming a within-group standard deviation of 16° and allowing for 5% loss to follow-up. Participants randomised to the Maxm Skate group will use the skate device and accompanying iOS App and sensors to complete rehabilitation exercises, as outlined in the *Maxm Skate Rehabilitation Guide*. Outcomes will be compared to those receiving standard rehabilitative care.

A blinded physiotherapist will evaluate functional outcomes preoperatively and at 2, 4, 6, 12, 26 and 52 weeks after TKA. The functional assessment will include measures of knee ROM, pain, isometric knee strength, balance and knee/thigh circumference. Limited measures will also be assessed at day 2 postoperatively by an alternate, unblinded physiotherapist. Clinical outcome measures will be administered preoperatively and at 6, 12 and 52 weeks postoperatively. An economic evaluation will be conducted and participants will be screened for adverse event occurrences from the time of consent to 12 months postoperatively.

**Discussion:**

This RCT will be the first to investigate the safety, efficacy and cost-effectiveness of the home-based Maxm Skate Rehabilitation program, in comparison to standard rehabilitative care following primary TKA.

**Trial registration:**

Australian New Zealand Clinical Trials Registry, ACTRN12616001081404p. Registered on 11 August 2016.

**Electronic supplementary material:**

The online version of this article (10.1186/s13063-018-3102-9) contains supplementary material, which is available to authorized users.

## Background

Primary total knee arthroplasty (TKA) is an increasingly common surgical intervention used to alleviate pain and physical dysfunction associated with end-stage degenerative joint disease. Following surgery, patients experience lower-extremity muscle weakness and commonly require physical rehabilitation to enhance functional outcomes and overall recovery [1, 2]. While TKA is a successful procedure for many patients, up to 15–20% of patients remain unsatisfied [3, 4]. Limited postoperative range of motion (ROM), which is often associated with arthrofibrosis, may be one factor associated with patient dissatisfaction. The recorded incidence of arthrofibrosis after primary TKA varies in the literature, in the range of approximately 1–13% [4–6]. When it occurs, it is a significant cause of patient dissatisfaction associated with poor ROM and is a causative factor of revision TKA [5, 6]. Therefore, as limited ROM is an early indicator of potential arthrofibrosis, rehabilitation that enables ongoing flexion/extension exercises and ongoing collection of such progress, may improve early detection of arthrofibrosis and as such appropriate rehabilitative management before the need for revision surgery. In addition, it is well established that daily activities including climbing stairs and standing from a chair require 90–120° of flexion, kneeling and squatting 110–165°, 135° of flexion to lift from a bath and > 150° for yoga or gardening [7–9]. However, patients rarely achieve > 120° of flexion post TKA and 110° of flexion is an optimal goal for rehabilitation following TKA [9–11]. At present, there are no universally accepted or widely implemented clinical guidelines available to consistently structure patient rehabilitation following TKA [12–14]. Consequently, rehabilitative protocols are not standardised and regularly based on institution-, surgeon- or occasionally, patient-specific preferences [1, 2, 12]. This lack of uniformity in the mode of rehabilitation results in therapy of different types, frequency, intensity and duration across sites and countries [1, 2].

In Australia, rehabilitation most commonly provided is in an outpatient setting, on a one-to-one treatment basis [12]. Outpatient physiotherapy in a clinic-based setting is beneficial as a physiotherapist can monitor progress and modify therapy; however, such methods are resource-intensive and impose a significant cost burden [2]. Further concerns include patient transportation to clinics following surgery and accessibility to qualified rehabilitative specialists.

In 2016, the number of primary TKA procedures performed in Australia increased by 139.8% since 2003 and by 2.8% compared to 2015 [15]. Such trends suggest that TKA use will continue to rise, bringing concern of the sustainability and economic impact of one-to-one rehabilitation commonly employed. There is a need to determine whether such outpatient physiotherapy yields superior outcomes compared to less costly alternate forms such as group-based, home-based or tele-rehabilitation.

Several randomised controlled trials (RCT) have considered the influence of outpatient physiotherapy on functional outcomes following TKA. Mockford et al. [16] and Rajan et al. [17] both concluded no significant difference in ROM achieved between patients who did and did not receive outpatient physiotherapy 12 months after TKA. However, methodologically short-comings, and limited descriptions of the standard outpatient physiotherapy provided, caused a literature reviewer to suggest that conclusions from these studies were not supported [2]. A methodologically robust RCT found that one-to-one outpatient treatment provided over a six-week period did not provide superior self-reported or performance-based outcomes, compared to group-based or monitored home programs up to 12 months after TKA [18]. Furthermore, a recent meta-analysis demonstrated no difference in knee ROM and short-term functional improvements between outpatient physiotherapy in a clinic-based setting and non-supervised home-based exercise regimens following discharge after TKA [19].

The common provision of one-to-one therapy is not currently supported over more economical home-based rehabilitative methods of which elicit similar self-reported, functional and performance-based outcomes [18, 20]. The current study will analyse a novel home-based rehabilitation program as compared to the standard rehabilitative care options available to patients. As detection of arthrofibrosis is typically first observed through reduced postoperative ROM, it is particularly important to this study to collect ROM data. Furthermore, early detection of poor ROM (three months) may enable implementation of appropriate rehabilitation before the requirement of manipulation under anaesthesia (MUA) or revision surgery.

The Maxm Skate device is a portable, lower-limb, postoperative and post-injury rehabilitation exercise device for individual use in a hospital or home-based setting. The Maxm Skate intends to facilitate rehabilitation and conditioning of the lower limb through graded therapeutic exercise with the aim to promote tissue healing, remodelling and strengthening. It allows the patient to perform strengthening exercises with minimal joint loading during their rehabilitation period.

The Maxm Skate package comprises the Skate device and rope (for assisted active motion and resistance training), sensors, iOS Application (App) and website. The Skate device is accompanied by two sensors and a mobile App which are designed to provide real-time, objective data on exercise and rehabilitation progress, in particular ROM following each home-based exercise therapy session. This sensor technology also enables the clinician to monitor accurate compliance and ROM data (Flexion and Extension) remotely.

This clinical trial is the first to compare the safety, efficacy and cost-effectiveness of the Maxm Skate rehabilitation package to standard rehabilitative care up to 12 months after TKA.

### Objectives

The primary objective of this RCT is to assess the ROM achieved by TKA patients whom received the Maxm Skate rehabilitation package compared with standard rehabilitative care, three months postoperatively.

Secondary objectives include the comparison of functional, clinical and performance-based outcome measures between the Maxm Skate and standard rehabilitative care groups at multiple times points, up to 12 months after TKA. An economic evaluation assessing the relative cost-effectiveness of the Maxm Skate rehabilitation package compared to standard care will be conducted; patients will be screened for adverse event (AE) occurrences and complications from the time of consent to one year postoperatively.

## Methods/Design

This is a single-blinded RCT comparing the safety, efficacy and cost-effectiveness of the Maxm Skate Rehabilitation Program to standard rehabilitative care following TKA.

### Study setting

The study will be coordinated by the International Musculoskeletal Research Institute Inc. at the study site, Flinders Private Hospital (FPH), Bedford Park, South Australia.

### Recruitment and selection criteria

Consecutive patients consented for elective primary TKA at a single orthopaedic surgeon’s private clinic will be screened for eligibility. The treating surgeon (MGL) will screen all patients against the selection criteria and document reasons for ineligibility in the patient screening log. Study inclusion criteria include:

1. The patient requires a primary TKA due to non-inflammatory degenerative joint disease (e.g. osteoarthritis and traumatic arthritis) or inflammatory joint disease (e.g. rheumatoid arthritis).
2. The patient must understand the conditions of the study and be willing and able to provide written informed consent.
3. The patient is a skeletally mature man or a non-pregnant woman, aged ≥ 30 years.
4. The patient agrees to comply with the specified preoperative and postoperative study requirements.

Study exclusion criteria include:

1. The patient has an emotional or neurological condition that would pre-empt their ability to participate in the study including mental illness, intellectual disability and drug or alcohol abuse.
2. Any patient who is unable to meet the requirements of the use of the Maxm Skate Rehabilitation Device.
3. The patient is unable to perform home exercise program without supervision or assistance.

A clinical nurse or authorised delegate will invite eligible patients to come in to discuss the study. Patients will be informed of the study purpose and the potential risks and benefits known, or that can be reasonably predicted, as outlined in the Participant Information Sheet.

The patient will then be given the opportunity to ask any clarifying questions and invited to complete the consent form, indicating their understanding of the study and consent for participation. Following consent, participants will be randomised and baseline measures obtained. The randomisation number will be used to provide anonymous identification of the participant on study documents from there forth.

### Randomisation and blinding

The randomisation schedule will be prepared using numbered and sealed, opaque envelopes. Participants will be randomly allocated in a 1:1 ratio to either the ‘Maxm Skate Rehabilitation Program’ or the ‘Standard Rehabilitative Care’ group. Block randomisation in groups of four will be used to ensure balance in the two groups. Randomisation will also be stratified by gender to ensure balance between groups for each gender. We note that the number of women undergoing TKA for osteoarthritis has been shown to be greater in Australia [21]. In addition, studies have demonstrated that there are differences between men and women in muscle and physical function recovery after TKA [22, 23].

The study will be single-blinded with blinding of study investigators but not participants. Participant randomisation to either Maxm Skate or Standard Care will be performed by the unblinded Study Coordinator, who will hold the treatment allocation code in confidence. The statistician will be blinded to the treatment allocation code when performing all analyses. Functional evaluations and performance tests will be conducted by a trained physiotherapist (SWH) who will be blinded to group allocation. This blinded physiotherapist will conduct all functional evaluations, excluding postoperative day 2, due to practical constraints. Non-blinded ward-based physiotherapists will conduct the day 2 assessments, as well as the preoperative Maxm Skate education session for participants allocated to the Maxm Skate group.

Due to the nature of the intervention there is a potential for participants to disclose, or physiotherapist to gauge, which group the participants are assigned. Participants will be educated and instructed not to advise the physiotherapist or treating surgeon of their group assignment at the time of their consent and randomisation with the unblinded Study Coordinator. However, if participants are concerned about their rehabilitative progress and feel it is necessary to discuss this with their treating surgeon, they will be able to do so. The breaking of blinding will be collected and reviewed during data analysis.

### Interventions

All participants will undergo primary TKA at Flinders Private Hospital. All hospital TKA patients are invited to attend a ‘Joint replacement information session’ which provides further education and the opportunity for physiotherapy and nursing staff to address patient queries. An exercise booklet is issued describing the postoperative exercises and patients are asked to familiarize themselves with the exercises.

#### Maxm Skate rehabilitation program

Participants randomised to the Maxm Skate rehabilitation program will be required to use the skate device and accompanying iOS App and sensors to complete rehabilitation exercises, as outlined in the *Maxm Skate Rehabilitation Guide* (see Additional file 1). Participants will be asked to download the Maxm Skate App on an iOS device, which will be provided if a personal device is not available.

Clinical data will be collected via the App aligns with the exercises outlined in the rehabilitation guide, including exercises completed, number of repetitions, length of time completing exercise and outcomes achieved. Data collected via the App are stored in the Google Firestore cloud database and within the iOS device itself.

##### Preoperative phase

Participants will attend a preoperative device education session where they will be introduced to the Maxm Skate program by a physiotherapist. Participants will be given a description of the functionality, set up and safe usage of the Maxm Skate device and sensor, and educated on the Maxm Skate exercise program. Following this presentation, participants will be issued with a rehabilitation guide to familiarise themselves with the postoperative exercise program and given the opportunity to trial the skate package.

##### Postoperative inpatient phase

In the inpatient acute setting, the Maxm Skate group will receive the Maxm Skate in addition to standard care as outlined above for the Standard Care group. The Maxm Skate group may access inpatient rehabilitation if clinically indicated.

##### Postoperative outpatient phase

Following the inpatient program, participants in the Maxm Skate program will only receive rehabilitation provided through the Maxm Skate program, described in the *Maxm Skate Rehabilitation Guide* (see Additional file 1). This program includes functional assessment checks at two weeks and six weeks at the participant’s home. This will ensure participants are fit to progress through from Stage One to Stage Two and from Stage Two to Stage Three of the program. Additional functional checks will be conducted if required, as assessed by hospital staff at discharge from hospital. Furthermore, the checks will allow early identification of participants who are not advancing and may require additional rehabilitative support.

Participants that are identified as requiring additional physiotherapy will receive such treatment, as required. Participant functional progress will also be assessed by the orthopaedic surgeon at the six-week orthopaedic follow-up appointment. Any additional outpatient physiotherapy received by a participant randomised to the Maxm Skate program will be permitted and recorded in the participant study file.

#### Standard rehabilitative care

Inpatient physiotherapy care provided to patients in hospital will include undertaking the following standard care exercises: ankle pumps; static quadriceps; supine knee flexion; inner range quadriceps; straight leg raise; passive knee extension; seated assisted knee flexion; and active knee extension in sitting. Once discharged from hospital, physiotherapy follow-up varies on an individual basis. The patient is provided four patient-specific options which are described in the *Outpatient Standard Care Physiotherapy Protocol* (*FPH*) (see Additional file 2).

### Study outcomes and participant timeline

**Fig. 1:**
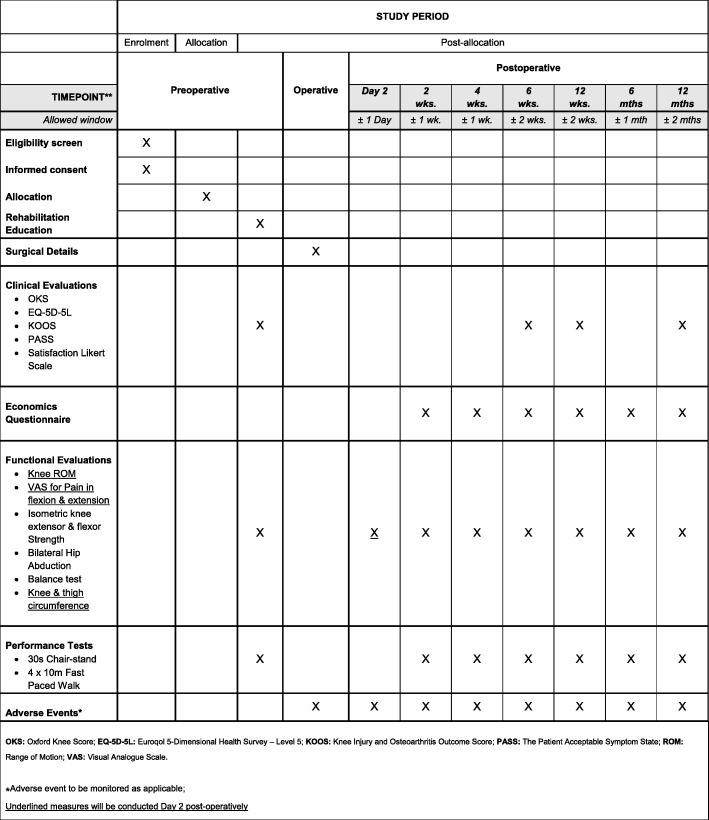
Schedule of events

The primary objective is ROM achieved at three months after TKA. Secondary outcomes include patient-reported outcomes, costs and functional evaluations which will be collected at multiple time-points up to 12 months after TKA. Patients will be screened for AE occurrences and complications from the time of consent to one year postoperatively. The study assessment schedule in outlined in Fig. 1.

#### Baseline and operative data

Demographic details, knee pathology and medical history will be collected preoperatively by the clinical nurse or research delegate.

In order to limit any potential variability attributed to the prosthetic device, a single total knee prosthesis, the Advanced Coated System ACS® Fixed Bearing System (Oceania Orthopaedics Pty. Ltd.) will be implanted in all study participants by a single orthopaedic surgeon (MGL). The medical device will be implanted using a medial para-patella surgical approach, using both a cemented and cementless technique depending on patient characteristics and surgeon discretion. A midline skin incision will be conducted for all patients and device-specific instrumentation will be used.

#### Patient-reported outcome measures

Patient-reported outcome measures will be assessed for each group preoperatively and postoperatively at six weeks, 12 weeks and 12 months. The Oxford Knee Score (OKS) [24] and Knee Injury and Osteoarthritis Outcome Score (KOOS) [25] will be used to assess knee-specific symptoms, pain and function, while the Euroqol 5 Dimensional Health Survey – Level 5 (EQ-5D-5L) [26] will be used to survey health status. The Patient Acceptable Symptom State (PASS) [27] will be used to assess whether the patient feels that they are at a satisfactory state. Lastly, level of satisfaction with the outpatient rehabilitation received will be measured using a 5-point Likert scale as follows: ‘very unsatisfied’; ‘unsatisfied’; ‘neither satisfied nor unsatisfied’; ‘satisfied’; ‘very satisfied’.

Participants in the Maxm Skate group will also be required to complete a short questionnaire regarding their participation in the Maxm rehabilitation program. This form will be administered at a visit with the Study Coordinator either before or after each functional evaluation with the blinded physiotherapist.

#### Functional evaluations

**Fig. 2:**
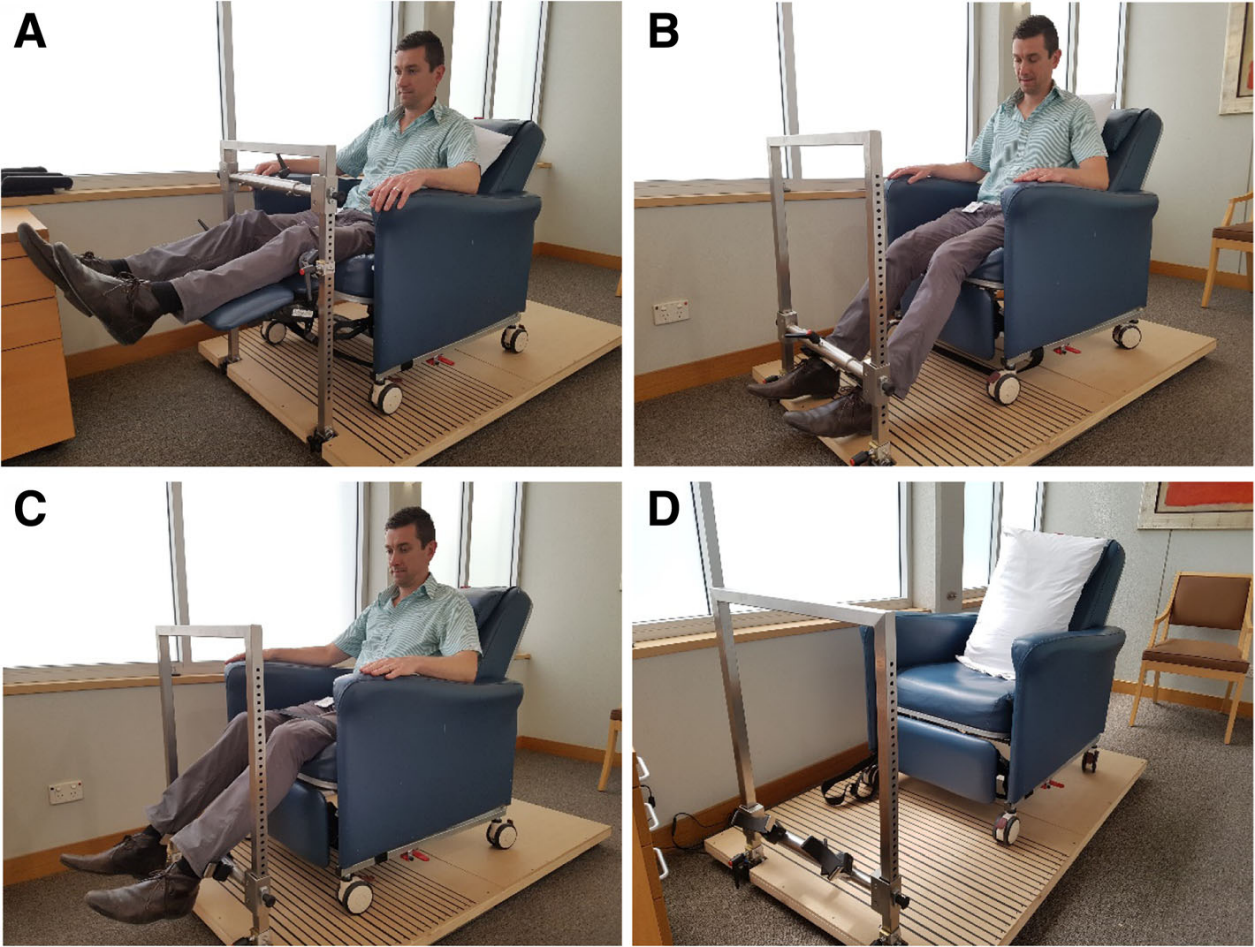
KangaTech set-up. **a** Hip abduction: for bilateral hip abduction testing, the participant will also be seated, with the knee flexed at 15° and lumbo-pelvic-hip complex flexed at 60°. The dynamometry pad will be positioned at the level of the lateral femoral condyles. **b** Knee extension: unilateral knee extension performed with participants seated, their knee at 45° flexion and lumbo-pelvic-hip complex at 60° flexion. The dynamometry pad will be positioned at the level of the lateral malleolus at right angles to the shank/tibia. Each thigh will be secured to the seat with a belt above the level of the surgical incision (> 10 cm above the supra patella border). **c** Knee flexion: unilateral knee extension performed with participants seated, their knee at 45° flexion and lumbo-pelvic-hip complex at 60° flexion. The dynamometry pad will be positioned at the level of the lateral malleolus at right angles to the shank/tibia. Each thigh will be secured to the seat with a belt above the level of the surgical incision (> 10 cm above the supra patella border). **d** Set-up alone at Flinders Private Hospital for use in this study

The following functional evaluations will be performed by a blinded physiotherapist preoperatively and at 2, 4, 6, 12, 26 and 52 weeks after TKA. Some measures will also be assessed at day 2 postoperatively; however, these measures will be taken by an unblinded ward physiotherapist. Functional evaulations are illustrated in Fig. 2.

##### Joint range of motion and the Visual Analogue Scale for Pain in flexion and extension

In a seated position, active joint ROM in flexion and extension will be measured using a standard goniometer. Furthermore, pain at end-of-range flexion and extension will be measured using the Visual Analogue Scale (VAS) for Pain. Recovery in knee ROM has been found to plateau 12 months after TKA, therefore end-of-study has been selected to be 12 months postoperatively [28]. Both measures will also be assessed at day 2 after TKA.

##### Isometric knee extensor strength, flexor strength and bilateral hip abduction

Hand-held isometric [29–32] and isokinetic [30, 31, 33, 34] dynamometry has previously been used to safely and effectively measure maximal quadricep and hamstring muscle strength in OA and TKR populations. In the present study, a customised fixed-frame portable dynamometry hardware and software system (KangaTech, Melbourne, Australia) will be used to measure maximum voluntary isometric strength of the quadriceps, hamstring and hip abductor muscle groups of each participant.

Maximum voluntary isometric contraction (MVIC) will be tested for the hip abductors, knee extensors and knee flexors in a seated position to maximise participant comfort. Unilateral knee extension and flexion tests will be performed with participants in a seated position with their knee at 45° of flexion and with their lumbo-pelvic-hip complex at 60° of flexion. The dynamometry pad will be positioned at the level of the lateral malleolus at right angles to the shank/tibia. Each thigh will be secured to the seat with a belt above the level of the surgical incision (> 10 cm above the supra patella border). For bilateral hip abduction testing, participants will also be seated, with the knee flexed at 15° and lumbo-pelvic-hip complex flexed at 60°. The dynamometry pad will be positioned at the level of the lateral femoral condyles.

Participants will perform each test in a randomly selected order after having had three gradually ramped practice/warm-up trials. During these trials, participants will be asked to produce an isometric contraction at 20%, 50% and 80% of their perceived maximum effort for 3 s. Participants experiencing any discomfort that is unusual and/or with an associated pain score of > 3/10 (measured by the VAS – Pain) above their resting level of pain will be asked not to proceed beyond this level of force or effort. In this case, the pain limited MVIC will be recorded.

Participants that warm up without the forementioned symptoms will proceed to 2 × 5 s MVIC test trials for each test. Participants will again be advised to limit force in the presence of discomfort. Time allowed for recovery between all tests will be 20 s.

##### Balance test, knee circumference and thigh circumference

This balance test will be performed in a single-leg standing position, with eyes open. The ability to maintain balance in this position will be recorded in seconds.

The participant’s knee and thigh circumference will be measured using a regular tape measure. The measurements will be taken at two points, specifically at the superior pole of the patella and 15 cm above the superior pole of the patella. Both measures will also be assessed at day 2 after TKA.

##### Clinical performance tests

Clinical performance tests, including the 30-s Chair-Stand Test and the 4 × 10 m Fast Paced Walk Test, will be performed in accordance with the Osteoarthritis Research Society International (OARSI) Report guidelines [35]. The tests will be conducted preoperatively and at 2, 4, 6 and 12 weeks, 6 and 12 months postoperatively.

#### Economic evaluation

An economic evaluation assessing the relative cost-effectiveness of the Maxm Skate rehabilitation device compared to standard care will be conducted.

This analysis will take the form of an economic evaluation comparing the relative cost-effectiveness analysis of the Maxm Skate rehabilitation device to standard care using data collected during the trial. The primary outcome will be the incremental cost per unit increase in knee ROM while a secondary outcome will be the incremental cost per quality-adjusted life year (QALY) gained over the period of the primary study and based on the EQ-5D-5 L. As an Australian scoring algorithm for the EQ-5D-5 L is not yet available, the UK value set and scoring algorithm [36] will be used to convert the individual responses to the EQ-5D-5 L at each time point. Resource use associated with each trial arm will be combined with unit costs for these resources in order to estimate total costs in the trial. Costs will be estimated from a societal perspective (to include costs borne by private hospitals and insurance providers, private patients and social care service providers). Resource use data will include a number of tests, treatments and investigations undertaken during in-hospital visits, the frequency and duration of in-hospital admissions, over the counter medication, lost productivity, social care services, as well as quantity and type of consumables and equipment used. Resource use data to capture patient-specific costs incurred will only be collected after discharge, using a resource use questionnaire that will be administered at 2, 4, 6, 12, 26 and 52 weeks. Resource unit costs will be derived from hospital finance departments and supplemented, where necessary, by information from published source including the Australian Bureau of Statistics and Australian Refined Diagnosis Related Groups (AR-DRG) cost weights. Confidence intervals will be presented around the incremental cost-effectiveness ratios and cost-effectiveness acceptability curves for varying threshold values of cost-effectiveness will also be presented [37]. An assessment of the sensitivity of the results obtained to variation in measured resource use, effectiveness and/or unit costs will be undertaken using appropriate deterministic and probabilistic sensitivity analyses [38].

### Study duration

The study accrual period will be approximately 12 months. Each individual will be followed for 12 months.

### Sample size

A sample size calculation based on achieving 90% power at two-tailed type 1 error rate of 5% identified that 110 participants (55 participants per group) would be required to detect 10° of difference in ROM between the Maxm Skate program and standard care groups at three months after TKA assuming a within-group SD of 16°. The clinically significant difference in ROM of 10° and SD was estimated from parameters described by Mockford et al. [16], in which the effect of a physiotherapy regimen on ROM was measured over a one-year, post-TKA follow-up period. Furthermore, it is well established that daily activities, including climbing stairs and standing from a chair, require 90–120° of flexion, kneeling and squatting 110–165°, and 135° of flexion to lift from a bath [7–9]. However, patients rarely achieve > 120° of flexion post TKA and 110° of flexion is an optimal goal for rehabilitation following TKA [9–11]. Based on such awareness of requirements for daily activities, a 10° difference in ROM may delineate between the ability to climb stairs or kneel down in TKA patients.

In order to account for a potential 5% loss to follow-up, a total sample of 116 participants (58 per group) will be recruited.

### Statistical methods

All analyses will be conducted on an intention-to-treat basis and results reported in accordance with CONSORT guidelines [39]. The primary endpoint is the ROM at three months and differences between groups will be assessed using an independent two-sample t-test with log-transformation of the outcome, if necessary, in order to meet the assumptions of normality. If normality is not achieved using transformation, then the Mann–Whitney U test will be used. Differences between groups for the clinical performance tests (30-s Chair-stand and 4 × 10 m Fast Paced Walk Test) and data collected on scales such as the OKS will also be assessed using a two-sample t-test or Wilcoxon Rank sum test if normality is not achieved. Baseline characteristics, including ROM, will be accounted for in a sensitivity analysis using mixed effects models that will also account for any missing data. Where > 10% of data are missing, multiple imputation using chained equations will also be used. Functional evaluations that are assessed at multiple time points will also be assessed using mixed effects models with an interaction term between group and visit used to determine differences in group effects across time. Two-sided hypothesis tests will be performed for each outcome with a type-1 error rate of alpha = 0.05 used for determining statistical significance.

### Assessment of safety and criteria for study discontinuation

The management of AEs that occur during this clinical trial will be in accordance with the guidelines of the approving Human Research Ethics Committee.

A safety analysis will be performed by a safety monitoring board that includes an independent orthopaedic field expert when 50% of the participants (58) reach the primary endpoint, at 12 weeks after TKA.

The primary criteria for discontinuation of the study will be poor outcomes, indicated by ROM. If > 18 participants (30%) in the experimental group (MAXM Skate program) present a ROM of < 90° at six weeks after TKA [40], the study will be discontinued. ROM assessments performed at six weeks will be conducted by both the study Physiotherapist at the six-week functional follow-up visit (specific to the study), as well as the treating orthopaedic surgeon at the routine six-week follow-up visit (routine clinical care at six weeks).

### Data management

Clinical study data will be recorded directly on the Case Report Form (CRF) and will be completed for each participant enrolled into the clinical study. The Principal Investigator or nominated Co-Investigator will review, approve and sign/date each completed CRF series, attesting to the accuracy and authenticity of the data entered.

Clinical study records will be filed in the Clinical Study Master File in an organised way that will facilitate management of the clinical study, audit and inspection. The files will be retained securely before archive and then archived for a period of not less than 15 years to allow for audit and inspection by regulatory authorities upon request.

The Principal Investigator will permit direct access of the study monitors, the appropriate Human Research Ethics Committee and appropriate regulatory authorities to the study data when required.

### Ethics and dissemination

This study received ethical approval from The Bellberry Human Research Ethics Committee on 24 September 2018. Any approved amendments made to the study protocol will be updated on the trial registry and the Therapeutic Goods Administration Clinical Trial Notification record, if appropriate.

Throughout the trial, investigators will endeavour to present preliminary findings at national and international orthopaedic conferences. At the conclusion of the trial, results will be prepared for presentation and publication in scientific journals.

## Discussion

With the increasing demand for TKA, further research must explore safe, optimal, cost-effective alternatives for physical rehabilitation following TKA. This single-blinded RCT will compare safety, efficacy and cost-effectiveness of a novel home-based approach, the Maxm Skate rehabilitation program to standard rehabilitative care following TKA.

### Study limitations

As is well discussed in the literature, there is potential for single-centre studies to have limited external validity. In the case of the present study, this may be considered a limitation given the enrolment of privately insured patients and the rehabilitation pathways offered to them in isolation, as compared to the rehabilitation pathways that may be available or typically selected in public healthcare centres. As such, in this study we are only capable of capturing the postoperative rehabilitation pathways that are routinely selected at Flinders Private Hospital. Interestingly, in Australia the mean discharge of privately insured TKA and THA patients to inpatient rehabilitation facilities is 40.1%, compared to 20% in public patients [41]. As such, this study will capture the frequency, length and type of rehabilitation the study participants receive, in the hopes of improving transparency and generalisability of care. In addition, the study will also be recruiting a single surgeon’s patients, which once again may reduce generalisability across surgeons within and across centres. Despite this, in order to reduce any potential bias associated with a single surgeon’s patient cohort, the study orthopaedic surgeon is to be completely blinded to treatment allocation and, wherever possible, blinded to study participation altogether. Previous studies have addressed the heterogeneity of standard care options for rehabilitation between private hospitals in Australia [42] and this also presents in our study as a potential limitation. Further to this, there may also be a deal of heterogeneity across the Maxm Skate group participants, as despite their allocation and use of the Maxm Skate exercise program, should they feel it necessary to receive additional physiotherapy to assist in their recovery, they are able to do so. These variations in rehabilitative care are unavoidable to provide equal and consistent clinical care opportunities across all patients. In order to account for such heterogeneity in the rehabilitative pathway, a clear outline of the frequency, duration and type of treatment received by each participant will be documented at each follow-up visit with the Study Coordinator. Furthermore, at study conclusion, per-protocol analyses will be performed within the Maxm Skate group to distinguish any differences observed between participants receiving any physiotherapy care additional to the Maxm Skate exercise program. Such ‘as-treated’ grouping may assist in addressing the recognised limitation of participant receipt of additional care masking any potential benefit of the Maxm Skate exercise program. Despite the above recognised limitations of this study, we believe this RCT will provide sound evidence describing the potential differences observed over the course of different rehabilitative pathways of TKA patients in our centre.

## Trial status

This publication is based on the study protocol version 08, dated 15 October 2018. Participant recruitment is anticipated to commence in October 2018, with an approximate recruitment period of 12 months. Details of the Maxm Skate study team are listed in Table 1.

**Table 1:** Maxm Skate Study team

Name	Role on team	Affiliation
Professor Jeganath Krishnan	Principle Investigator	College of Medicine and Public Health, Flinders University, Adelaide, South Australia, Australia; The International Musculoskeletal Research Institute Inc., Adelaide, Australia
Dr Matthew G. Liptak	Co-investigator; treating Orthopaedic Surgeon	Orthopaedic SA, Adelaide, Australia
Annika Theodoulou	Clinical Trial Coordinator (pre-recruitment [study design and set-up])	College of Medicine and Public Health, Flinders University, Adelaide, South Australia, Australia; The International Musculoskeletal Research Institute Inc., Adelaide, Australia
Dr Kristen Georgiou	Clinical Trial Coordinator (during recruitment)	College of Medicine and Public Health, Flinders University, Adelaide, South Australia, Australia; The International Musculoskeletal Research Institute Inc., Adelaide, Australia
Scott W. Hinrichs	Co-investigator; blinded physiotherapist	Flinders Private Hospital, Adelaide, Australia
Dr Steve Saunders	Physiotherapist; development of Maxm Skate rehabilitation program	Adjunct researcher – University of South Australia Director – Saunders Sports and Spinal Science and Medical Coordinator – Adelaide Football Club
A/Prof Billingsley Kaambwa	Health Economist	Health Economics Unit, College of Medicine and Public Health Flinders University
Prof Richard J. Woodman	Biostatistician	Flinders Centre for Epidemiology and Biostatistics, College of Medicine, Flinders University

This protocol publication has been prepared on the basis of the Standard Protocol Items: Recommendations for Interventional Trials (SPIRIT) guidelines. This SPIRIT Checklist is available in the Additional file 3.

## Additional files

Additional file 1: Maxm Skate Rehabilitation Guide. (PDF 2897 kb)

Additional file 2: Outpatient Standard Care Physiotherapy Protocol (FPH). (DOCX 15 kb)

Additional file 3: SPIRIT Checklist. (DOC 123 kb)

## Abbreviations

AE: Adverse event
App: iOS Application
AR-DRG: Australian Bureau of Statistics and Australian Refined Diagnosis Related Groups
CRF: Case Report Form
EQ – 5D 5L: Euroqol 5 Dimensional Health Survey – Level 5
FPH: Flinders Private Hospital
iOS: iPhone Operating System
KOOS: Knee Injury and Osteoarthritis Outcome Score
MVIC: Maximum voluntary isometric contraction
OARSI: Osteoarthritis Research Society International
OKS: Oxford Knee Score
PASS: Patient Acceptable Symptom State
QALY: Quality-adjusted life year
RCT: Randomised controlled trial
ROM: Range of motion
TKA: Total knee arthroplasty
UK: United Kingdom
VAS – Pain: Visual Analogue Scale for Pain
