# Supplementary material for: The safety, efficacy and cost-effectiveness of the Maxm Skate, a lower limb rehabilitation device for use following total knee arthroplasty: study protocol for a randomised controlled trial
Source: Trials. 2019 Jan 10;20:36. doi: 10.1186/s13063-018-3102-9 (PMC6329189; doi:10.1186/s13063-018-3102-9)
Supplement: Supplementary file 1 — Maxm Skate Rehabilitation Guide. (PDF 2897 kb) [file 13063_2018_3102_MOESM1_ESM.pdf]

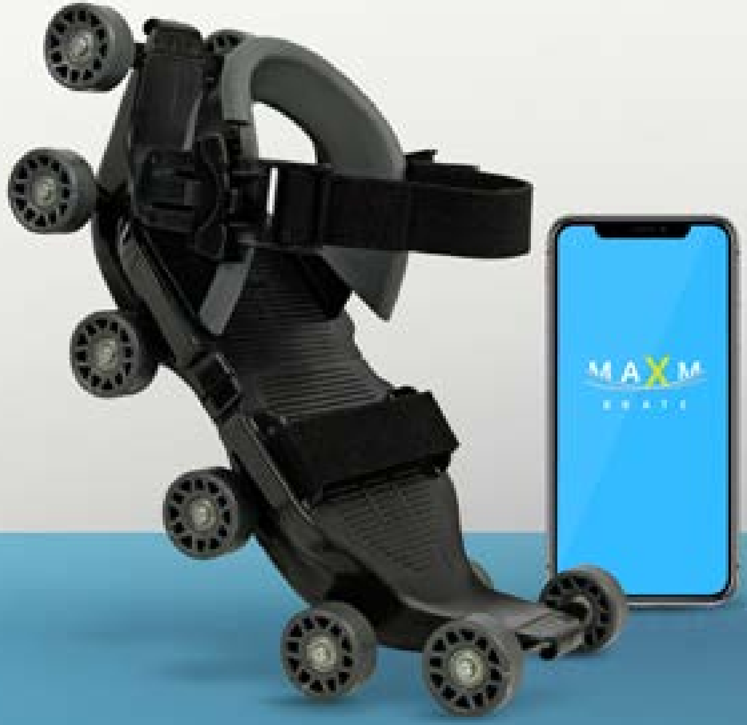

MAXM SKATE

REHABILITATION GUIDE

CLINICAL TRIAL EDITION

THIS DEVICE IS CURRENTLY UNDER CLINICAL TRIAL INVESTIGATION

MAXM SKATE

REHABILITATION GUIDE

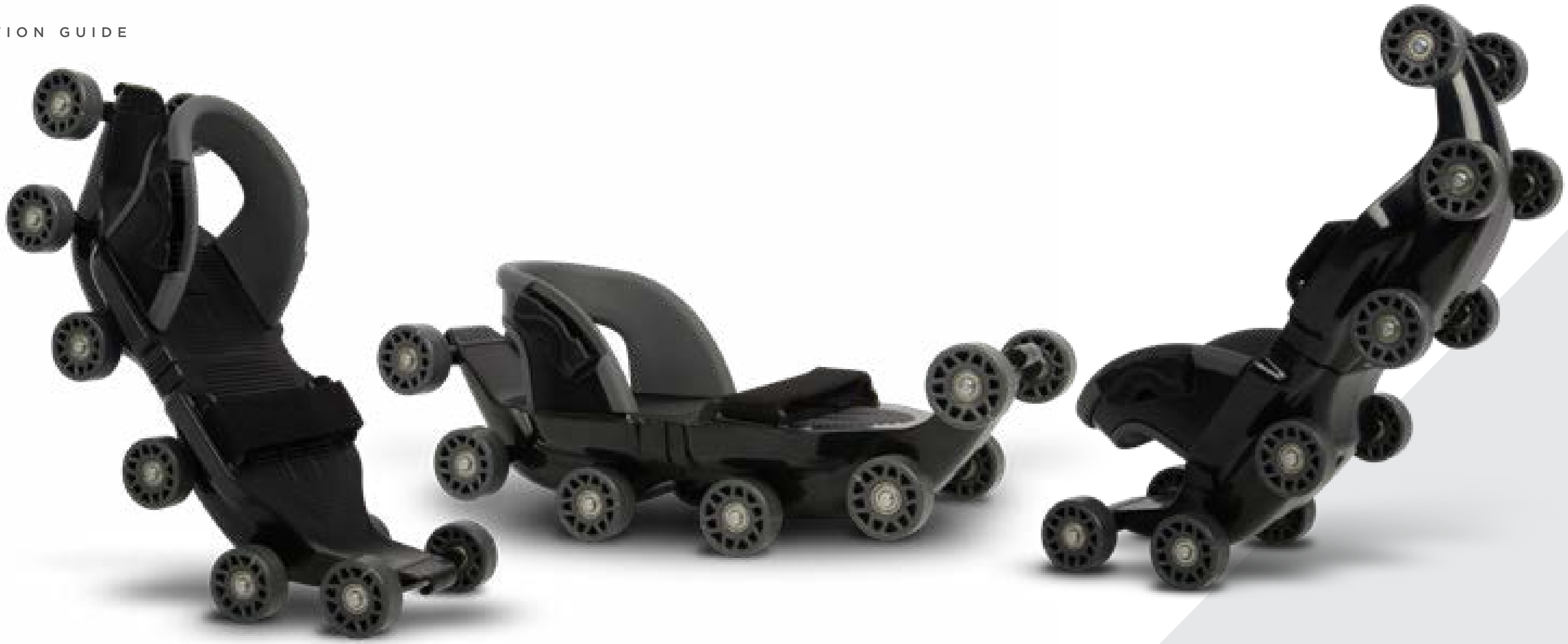

CONTENTS

STAGE 01	MOVEMENT	P06
STAGE 02	ACTIVE	P12
STAGE 03	EXTENSION	P18
STAGE 04	MAXIMUM	P24

© MAXM Skate Pty Ltd

© MAXM Skate Pty Ltd

MAXM SKATE

REHABILITATION GUIDE

The MAXM skate is a medical rehabilitation device designed for use following total knee replacement surgery. The effectiveness and safety of the device is currently under clinical trial investigation.

The MAXM Skate, Sensors, App and Exercise program are designed to provide real time, objective data on exercise and rehabilitation progress following an exercise therapy session at home. The MAXM exercise program has been divided into 4 stages. Incremental progressions are based on time and function.

MAXM TOTAL KNEE REPLACEMENT REHABILITATION PROGRAM

M — STAGE 1 MOVEMENT — DAYS 0-14 →

A — STAGE 2 ACTIVE — WEEKS 2-6 →

X — STAGE 3 EXTENSION — WEEKS 7-12 →

M — STAGE 4 MAXIMUM — WEEK 12

- Never stand while the Maxm Skate in attached to your foot.
- Only use the Maxm Skate as described in your information booklet and as indicated in this exercise booklet.
- Stop and seek advice if you are experiencing any increase in pain, particularly if not settling.
- If you have any questions, ask your hospital physiotherapist or call your surgeon's consulting rooms.

For further information and contact details please refer to maxmskate.com.aul

MAXM SKATE

REHABILITATION GUIDE

SESSION OUTLINE

(25 MINS)

Each daily Maxm rehabilitation session aims to promote healing, restore movement, increase muscle strength and restore function.

These sessions are progressed over the 4 stages of your rehabilitation (see diagram).
Maxm technology will keep you informed on how you are progressing during each component of each phase of your rehabilitation.

SKATE MOVEMENT | STRENGTH | FUNCTIONAL

© MAXM Skate Pty Ltd

EACH STAGE HAS 3 CATEGORIES OF EXERCISES.

SKATE/ MOVEMENT	STRENGTH	FUNCTIONAL
JOINT RANGE	MUSCLE STRENGTH	FUNCTION INTEGRATION
MUSCLE ACTIVATION	PROPRIOCEPTION	FITNESS
HEALING	BALANCE	LIFESTYLE

© MAXM Skate Pty Ltd

SENSOR SET UP

A. Turn on sensors
- white sensor flashes *red* when activated
- blue sensor flashes *green* when activated

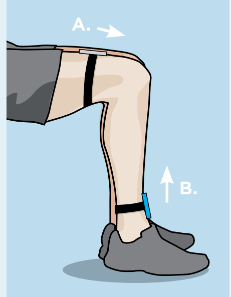

B. Place White static sensor 10cm above the top of your knee cap and secure strap

C. Place blue motion sensor 5cm above your ankle and secure strap

D. Turn on app
- *select stage*
- *select exercise*
- *sensor will now sync with app*
- *when synced all lights on sensors will stop flashing*

NB: BOTH ARROWS ON THE SENSORS MUST POINT TO THE KNEECAP.

M

MOVEMENT

STAGE 1
DAYS 0-14

© MAXM Skate Pty Ltd

M

MOVEMENT STAGE 1 - DAYS 0-14

RANGE OF MOTION AND WARM UP

MAXM SKATE

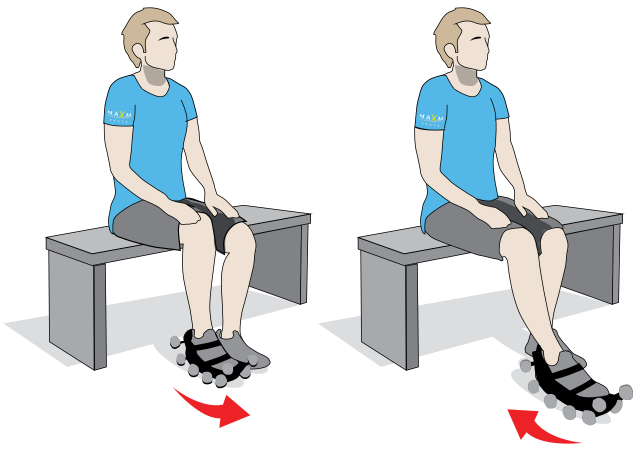

FLAT SKATE

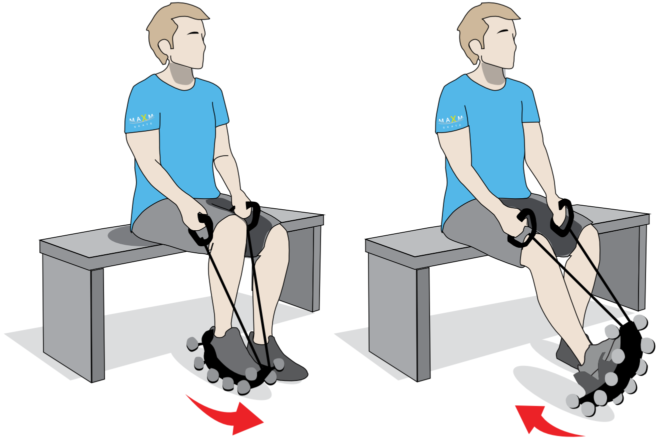

EXTENSION SKATE

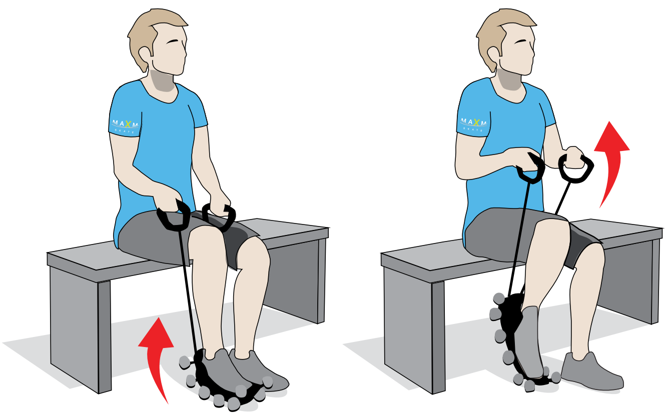

FLEXION SKATE

FLAT SKATE

INSTRUCTION

- In a seated position
- Glide skate forward and backward
- Keeping wheels flat on the floor at all times

FREQUENCY

- **3 sets x 40 reps**
- **Smooth, slow movement**
- **3 Sessions per day**

EXTENSION SKATE

INSTRUCTION

- In a seated position glide skate forward
- Engage quadriceps to further straighten knee
- Pull back on the MAXM strap to maximise stretch
- Hold for 2 seconds then release tension
- Return to start position & repeat

FREQUENCY

- **3 sets x 10 reps with 2 secs hold**
- **3 Sessions per day**

FLEXION SKATE

INSTRUCTION

- In a seated position place MAXM strap into back hook
- Glide skate forward then backwards
- Pull up on MAXM strap to maximise flexion (knee bend)
- Hold for 2 seconds
- Return to start position & repeat

FREQUENCY

- **3 sets x 10 reps with 2 secs hold**
- **3 Sessions per day**

© MAXM Skate Pty Ltd

MAXM SKATE

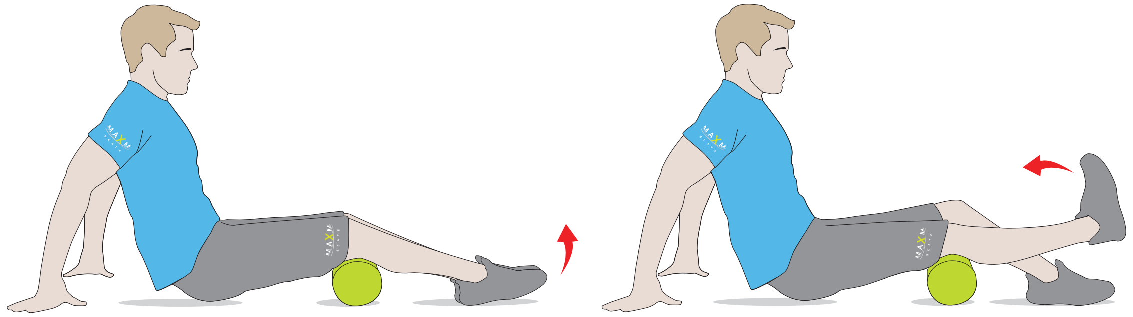

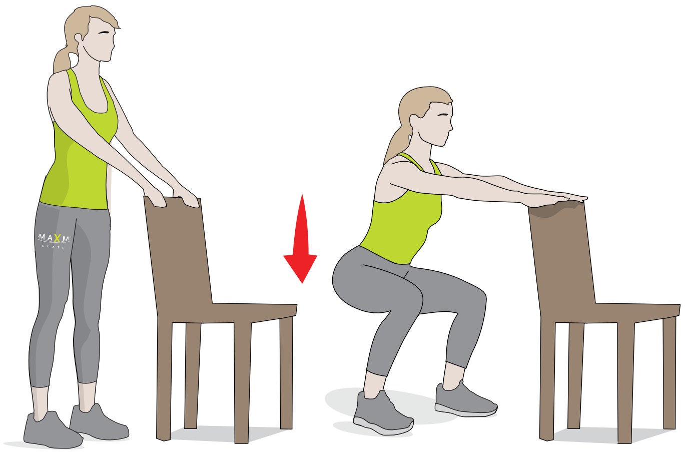

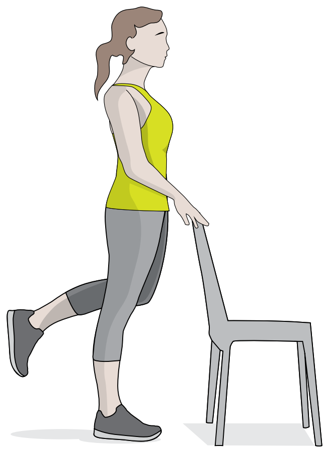

IRQ LONG SITTING

INSTRUCTION

- Pull toes back
- Squeeze inner quads/thigh
- Lift heel slowly, pressing back of thigh into towel
- Hold knee straight 2 seconds
- Lower heel slowly
- Relax & repeat

FREQUENCY

- **3 sets x 10 reps**
- **3 sessions per day**

nb: Quads/thigh muscles should fatigue

SUPPORTED DOUBLE LEG SQUAT

INSTRUCTION

- Stand facing a chair. Light finger pressure on chair can be used for balance
- Maintaining even pressure through both heels
- ¼ squat slowly & hold for 2 seconds
- Return to start position & repeat

FREQUENCY

- **3 sets x 10 reps**
- **3 sessions per day**

SUPPORTED SINGLE LEG BALANCE

INSTRUCTION

- Stand facing a chair with light finger pressure on chair for balance
- Balance on operated leg keeping equal pressure through heel and ball of big toe
- Keep pelvis level and knee over foot
- Hold for 10 seconds

FREQUENCY

- **3 sets x 10 secs hold**
- **3 sessions per day**

© MAXM Skate Pty Ltd

© MAXM Skate Pty Ltd

M

STRENGTH STAGE 1 - DAYS 0-14

CALF HIP AND HAMSTRING

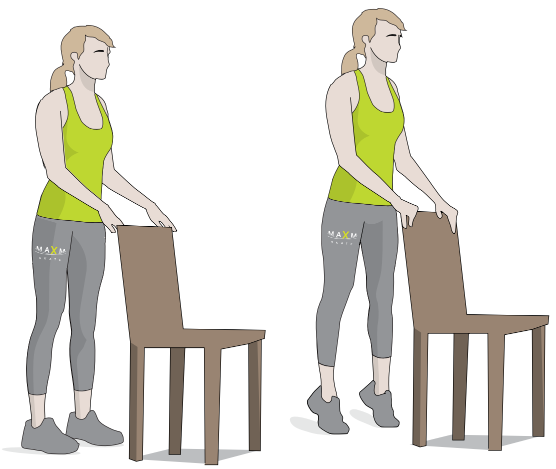

SUPPORTED STANDING HEEL RAISE

INSTRUCTION

- Face chair
- Stand on both legs with feet shoulder width apart touching support lightly with fingers
- Rise onto toes slowly, keeping pressure on ball of big toe
- Hold for 1 second, lower slowly to the floor & repeat

FREQUENCY

- 3 sets x 10 reps
- 3 sessions per day

nb: calf muscles should fatigue

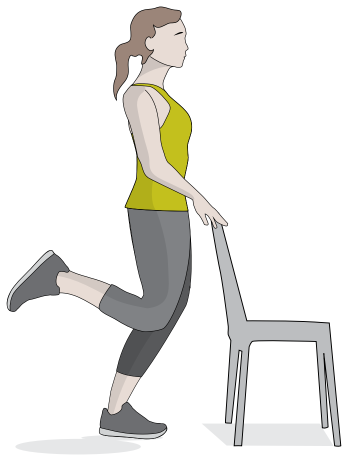

STANDING SUPPORTED HAMSTRING CURL

INSTRUCTION

- Standing in supported position
- Slowly bend operated knee as far as possible
- Hold for 1 second
- Slowly lower to floor & repeat

FREQUENCY

- 3 sets x 10 reps
- 3 sessions per day

nb: hamstrings should fatigue

M

FUNCTIONAL STAGE 1 - DAYS 0-14

FUNCTIONAL INTEGRATION

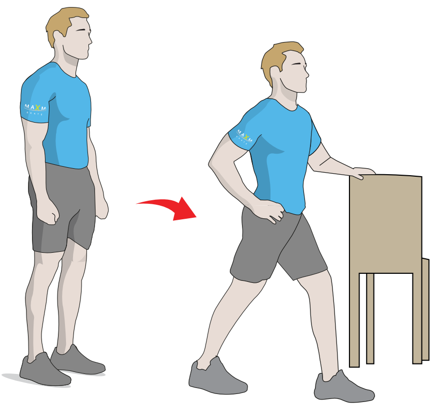

SUPPORTED MINI LUNGES

INSTRUCTION

- Stand with feet level using a chair as support
- Mini lunge on operated leg
- Hold for 1 second
- Push back to start position & repeat
- Perform exercise on non-operated leg

FREQUENCY

- 3 sets x 5 reps (each leg)
- 3 sessions per day

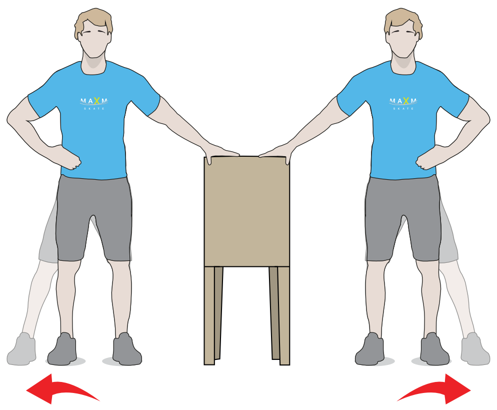

SUPPORTED SIDE STEP DRILL

INSTRUCTION

- Stand with feet level using a support
- Slowly step to the right side 10 cm over an imaginary object & return back to start position
- Slowly step to left side 10 cm over an imaginary object & return back to start position
- Repeat
- Repeat other direction, stepping to the left

FREQUENCY

- 3 sets x 10 reps (5 each direction)
- 3 sessions per day

WALKING WITH THE SENSORS ON FOR 5 MINUTES, 3 TIMES PER DAY.

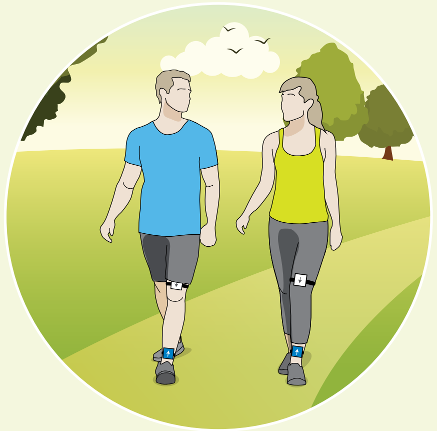

WALKING TARGET VOLUME GOALS

STEPS PER DAY

END WEEK 1	1,000 STEPS PER DAY
END WEEK 2	2,000 STEPS PER DAY

© MAXM Skate Pty Ltd

A

ACTIVE

STAGE 2
WEEKS 2-6

© MAXM Skate Pty Ltd

A | MOVEMENT STAGE 2 - WEEKS 2-6
RANGE OF MOTION AND WARM UP

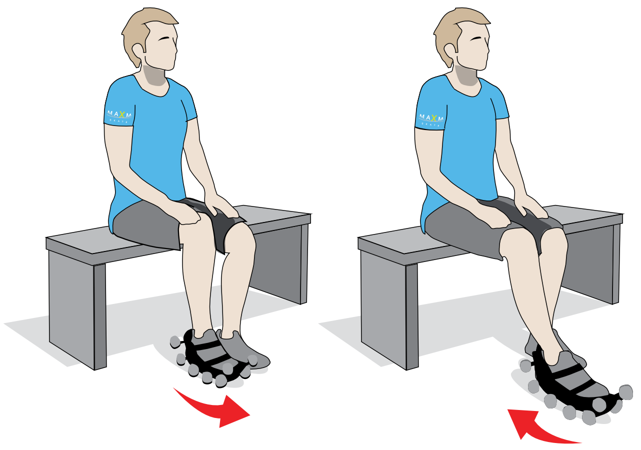

FLAT SKATE

INSTRUCTION

- In a seated position
- Glide skate forward and backward
- Keeping wheels flat on the floor at all times

FREQUENCY

- 3 sets x 40 reps
- Smooth movement gradually increasing speed
- 3 sessions per day

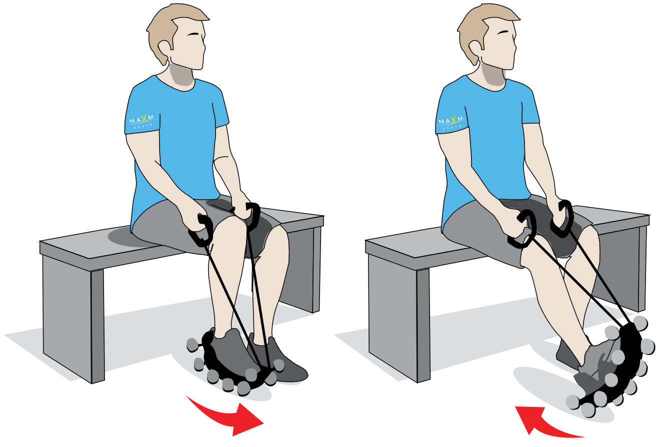

EXTENSION SKATE

INSTRUCTION

- In a seated position place MAXM strap into front hook & glide skate forward
- Engage quadriceps to further straighten knee
- Pull back on the MAXM strap to maximise stretch
- Hold for 2 seconds then release tension
- Return to start position & repeat

FREQUENCY

- 3 sets x 15 reps with 2 secs hold
- 3 sessions per day

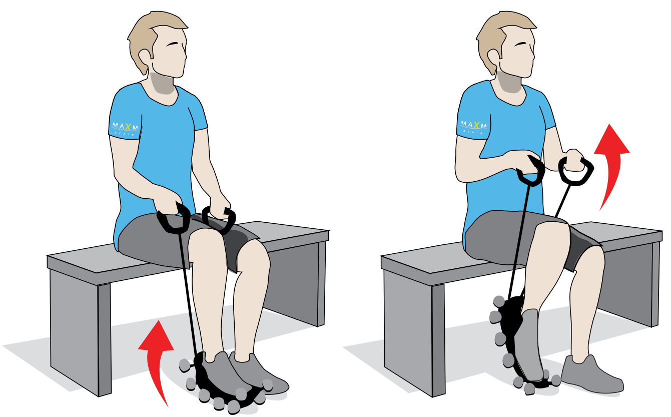

FLEXION SKATE

INSTRUCTION

- In a seated position place MAXM strap into back hook
- Glide skate forward then backwards
- Pull up on MAXM strap to maximise flexion (knee bend)
- Hold for 2 seconds
- Return to start position & repeat

FREQUENCY

- 3 sets x 15 reps with 2 secs hold
- 3 sessions per day

© MAXM Skate Pty Ltd

A | STRENGTH STAGE 2 - WEEKS 2-6
QUADRICEPS AND SINGLE LEG BALANCE

MAXM SKATE

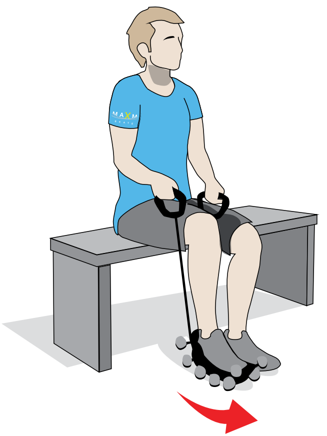

RESISTED EXTENSION SKATE

INSTRUCTION

- Place MAXM strap in back hook & bend knee to 90 degrees
- Provide resistance by pulling on MAXM strap and engage quadricep
- Slowly straighten knee smoothly against resistance as far as possible whilst skate remains flat on floor
- Hold for 2 seconds & maintain tension
- Slowly allowing knee to return to start position

FREQUENCY

- 3 sets x 10 reps
- 3 sessions per day

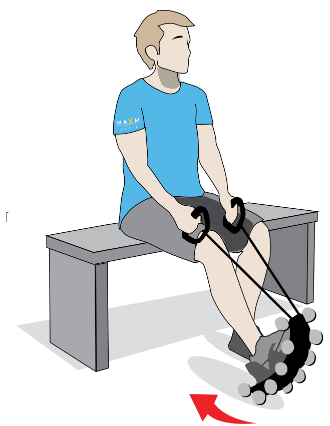

RESISTED FLEXION SKATE

INSTRUCTION

- Place MAXM strap in front hook,
- With no resistance straighten knee
- Pull on MAXM strap & engage hamstring
- Claw/pull heel smoothly against resistance until knee is maximally bent & hold for 2 seconds
- Maintain resistance while slowly allowing skate to return to start position
- Relax & repeat

FREQUENCY

- 3 sets x 10 reps
- 3 sessions per day

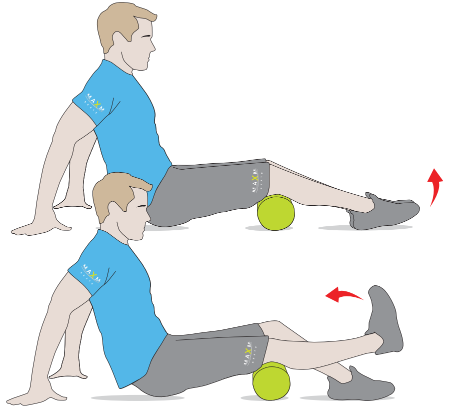

IRQ LONG SITTING

INSTRUCTION

- Pull back toes
- Squeeze inner quad/thigh
- Lift heel slowly, pressing back of thigh into towel
- Hold knee straight 2 seconds
- Lower heel
- Relax & repeat

FREQUENCY

- 3 sets x 15 reps
- 3 sessions per day

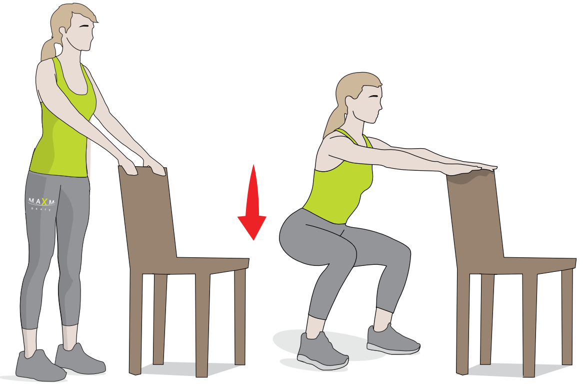

SUPPORTED DOUBLE LEG SQUAT

INSTRUCTION

- Stand facing a chair with light finger pressure on chair for balance
- Keeping weight through heels
- ¼ squat & hold for 2 seconds
- Return to start position & repeat

FREQUENCY

- 3 sets x 15 reps
- 3 sessions per day

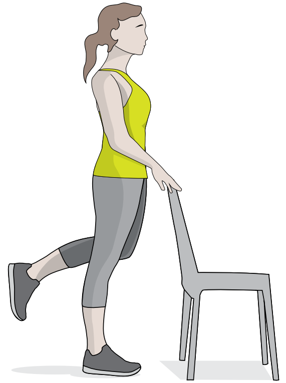

SUPPORTED SINGLE LEG BALANCE

INSTRUCTION

- Stand facing a chair with light finger pressure on chair for balance
- Balance on operated leg keeping equal pressure through heel and ball of big toe
- Keep pelvis level and knee over foot
- Hold for 10-30 seconds

FREQUENCY

- 3 sets x 10-30 second hold
- 3 sessions per day

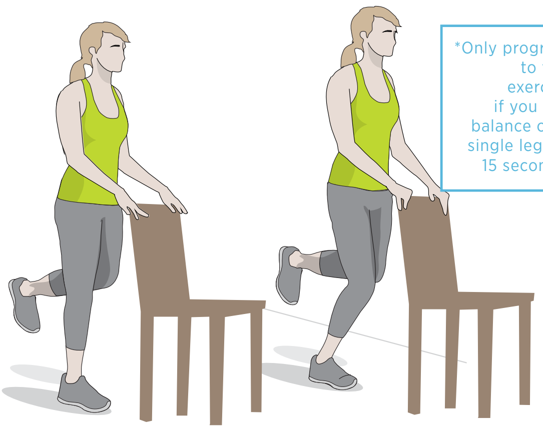

*Only progress to this exercise if you can balance on a single leg for 15 seconds.

SUPPORTED SINGLE LEG KNEE BEND

INSTRUCTION

- Stand facing chair with light finger touch for support
- Balance on operated leg
- Keep waist/belt line horizontal to the ground
- ¼ squat & hold for 2 seconds
- Return to start position & repeat

FREQUENCY

- 3 sets x 5 reps
- 3 sessions per day

© MAXM Skate Pty Ltd

A | STRENGTH STAGE 2 - WEEKS 2-6
CALF HIP AND HAMSTRING

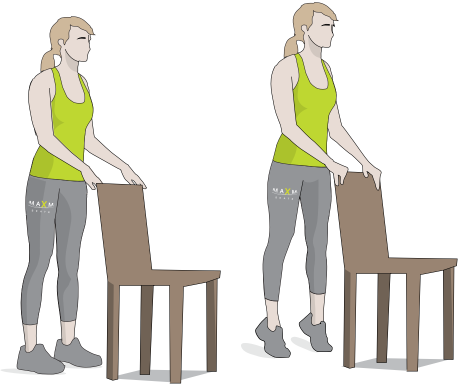

SUPPORTED STANDING HEEL RAISE

INSTRUCTION

- Face chair
- Stand on both legs with feet shoulder width apart touching support lightly with fingers
- Rise onto toes slowly, keeping pressure on ball of big toe
- Hold for 1 sec, lower slowly to the floor & repeat

FREQUENCY

- 3 sets x 15 reps
- 3 sessions per day

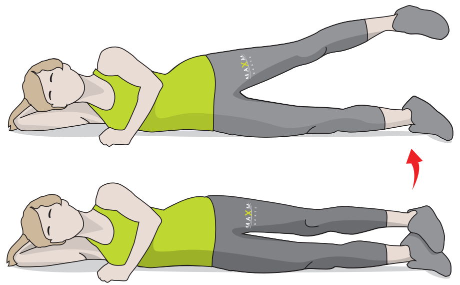

HIP ABDUCTION (SIDE LYING)

INSTRUCTION

- Lay on side with hips perpendicular to the floor
- Bend hips and knees slightly & rotate upper knee upwards turning hip out without moving pelvis
- Lift leg 20 cms & hold for 2 seconds
- Lower slowly, relax & repeat on opposite leg

FREQUENCY

- 3 sets x 5 reps (each leg)
- 3 sessions per day

nb: gluteal muscle on side of hip should fatigue

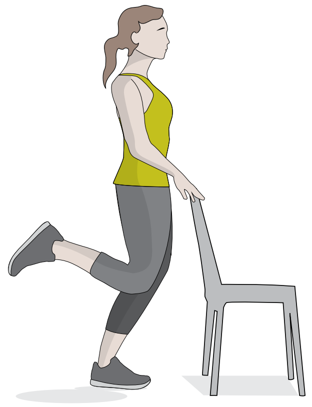

STANDING SUPPORTED HAMSTRING CURL

INSTRUCTION

- Standing in supported position
- Slowly bend operated knee as far as possible
- Hold for 1 second
- Slowly lower to the floor & repeat

FREQUENCY

- 3 sets x 10 reps
- 3 sessions per day

nb: Hamstrings should fatigue

A | FUNCTIONAL STAGE 2 - WEEKS 2-6
FUNCTIONAL INTEGRATION

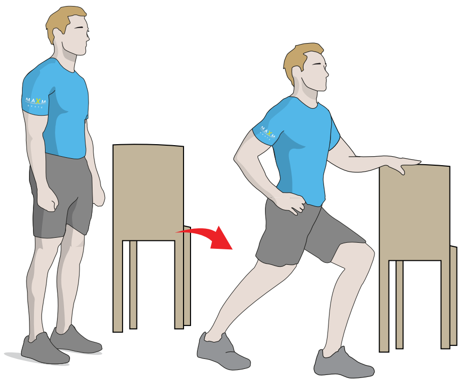

SUPPORTED LUNGES

INSTRUCTION

- Stand with feet level using a chair for support
- Slowly step forward 40 cms on operated leg
- Bend knee slowly 20 degrees & hold for 1 second
- Push back to start position & repeat whole set
- Perform exercise on non-operated leg

FREQUENCY

- 3 sets x 5 reps (each leg)
- 3 sessions per day

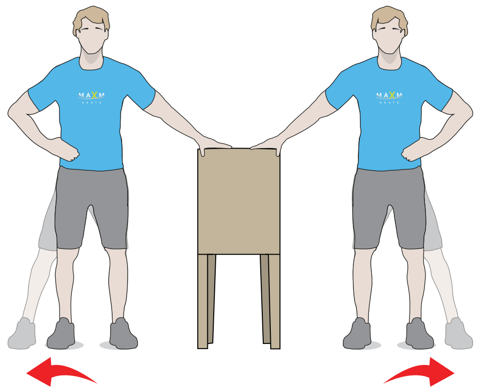

SUPPORTED SIDE STEP DRILL

INSTRUCTION

- Stand with feet level using a support
- Slowly step to the right side 10 cm over an imaginary object & return back to start position
- Slowly step to left side 10 cm over an imaginary object & repeat other direction
- Return back to start position & repeat

FREQUENCY

- 3 sets x 15 reps (5 each direction)
- 3 sessions per day

WALKING WITH THE SENSORS ON FOR 10 MINUTES, 3 TIMES PER DAY.

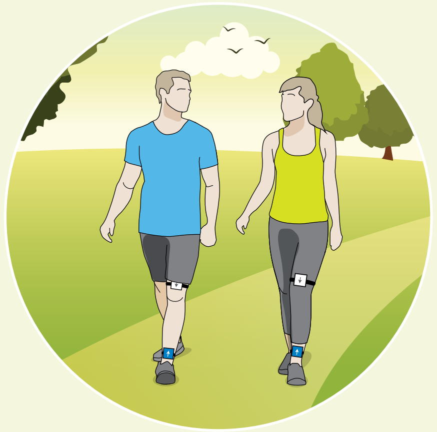

WALKING TARGET VOLUME GOALS
STEPS PER DAY

END WEEK 3	2,500 STEPS PER DAY
END WEEK 4	3,000 STEPS PER DAY
END WEEK 5	3,500 STEPS PER DAY
END WEEK 6	4,000 STEPS PER DAY

© MAXM Skate Pty Ltd

X

EXTENSION

STAGE 3
WEEKS 7-12

© MAXM Skate Pty Ltd

X MOVEMENT STAGE 3 - WEEKS 7-12

RANGE OF MOTION AND WARM UP

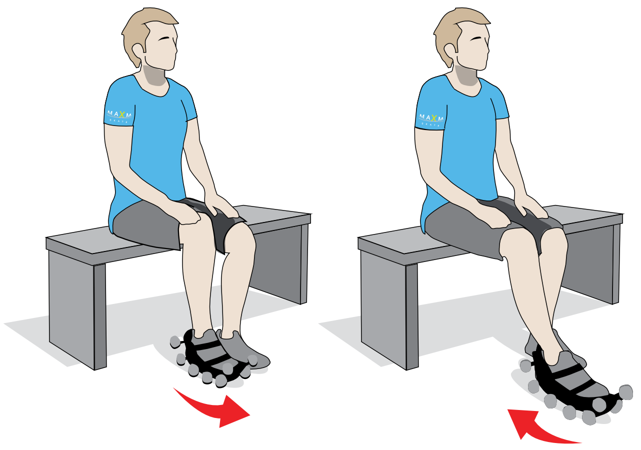

FLAT SKATE

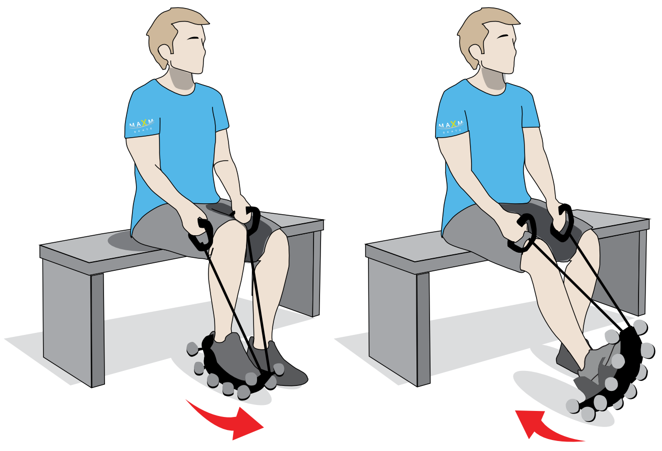

EXTENSION SKATE

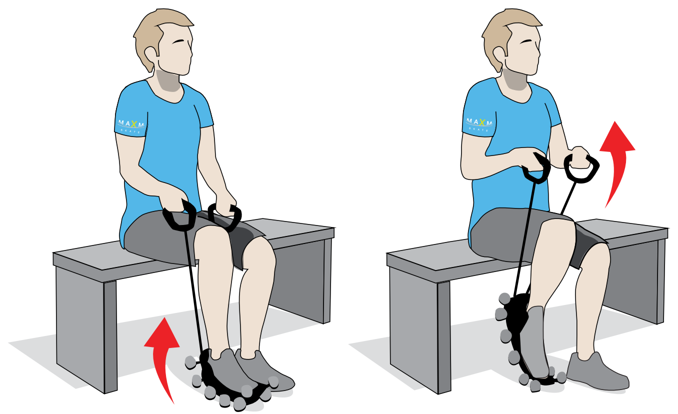

FLEXION SKATE

FLAT SKATE

INSTRUCTION

- In a seated position
- Glide skate forward and backward
- Keeping wheels flat on the floor at all times

FREQUENCY

- 3 sets x 40 reps
- Smooth movement, varying speed and range
- 3 sessions per day

EXTENSION SKATE

INSTRUCTION

- In a seated position place MAXM strap into front hook & glide skate forward
- Engage quadriceps to further straighten knee
- Pull back on the MAXM strap to maximise stretch
- Hold for 2 seconds then release tension
- Return to start position & repeat

FREQUENCY

- 3 sets x 20 reps with 2 secs hold
- 3 sessions per day

FLEXION SKATE

INSTRUCTION

- In a seated position place MAXM strap into back hook
- Glide skate forward then backwards
- Pull up on MAXM strap to maximise flexion (knee bend)
- Hold for 2 seconds
- Return to start position & repeat

FREQUENCY

- 3 sets x 20 reps with 2 secs hold
- 3 sessions per day

© MAXM Skate Pty Ltd

STRENGTH STAGE 3 - WEEKS 7-12

QUADRICEPS AND SINGLE LEG BALANCE

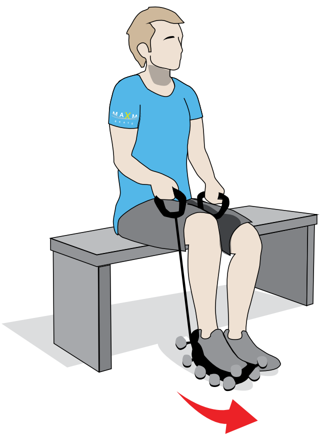

RESISTED EXTENSION SKATE

INSTRUCTION

- Place MAXM strap in back hook & bend knee to 90 degrees
- Provide resistance by pulling on MAXM strap & engage quadricep
- Slowly straighten knee smoothly against resistance as far as possible whilst skate remains flat on floor
- Hold for 2 seconds & maintain tension
- Slowly allowing knee to return to start position

FREQUENCY

- 3 sets x 20 reps
- 3 sessions per day

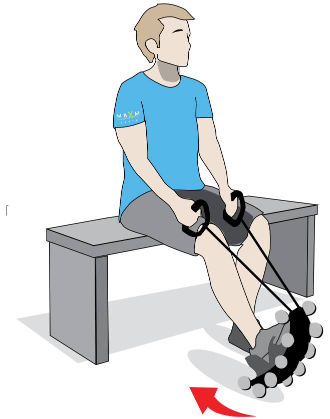

RESISTED FLEXION SKATE

INSTRUCTION

- Place MAXM strap in front hook,
- With no resistance straighten knee
- Pull on MAXM strap & engage hamstring
- Claw/pull heel smoothly against resistance until knee is maximally bent & hold for 2 seconds
- Maintain resistance while slowly allowing skate to return to start position
- Relax & repeat

FREQUENCY

- 3 sets x 20 reps
- 3 sessions per day

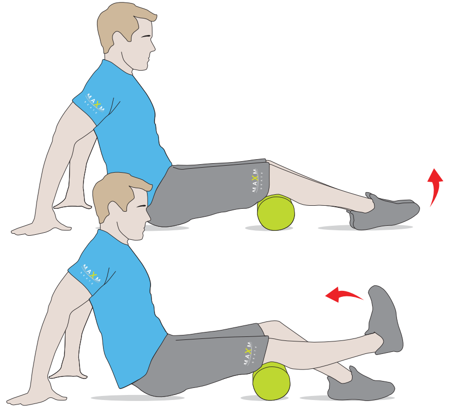

IRQ LONG SITTING

INSTRUCTION

- Pull back toes
- Squeeze inner quad/thigh
- Lift heel slowly, pressing back of thigh into towel
- Hold knee straight 2 seconds
- Lower heel
- Relax & repeat

FREQUENCY

- 3 sets x 20 reps
- 3 sessions per day

© MAXM Skate Pty Ltd

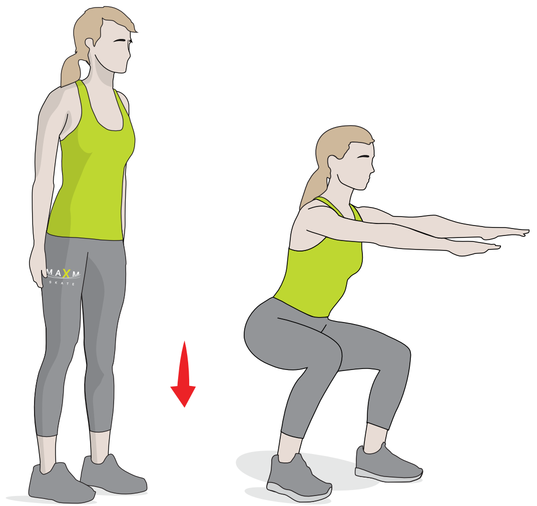

DOUBLE LEG SQUAT

INSTRUCTION

- Stand with feet hip width apart, hands on hips
- Maintain even weight through both heels and bend knees to lower pelvis 30 cms
- Hold for 2 seconds
- Return to start & repeat

FREQUENCY

- 3 sets x 15 reps
- 3 sessions per day

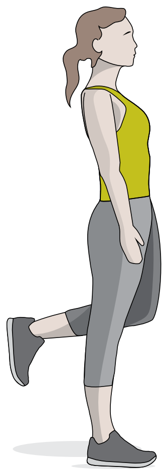

SINGLE LEG BALANCE

INSTRUCTION

- Stand on operated leg
- Balance keeping pelvis level with weight evenly distributed through heel and back of big toe
- Hold for 30 seconds
- Change legs

FREQUENCY

- 3 sets balancing for 10-30 secs (each leg)
- 3 sessions per day

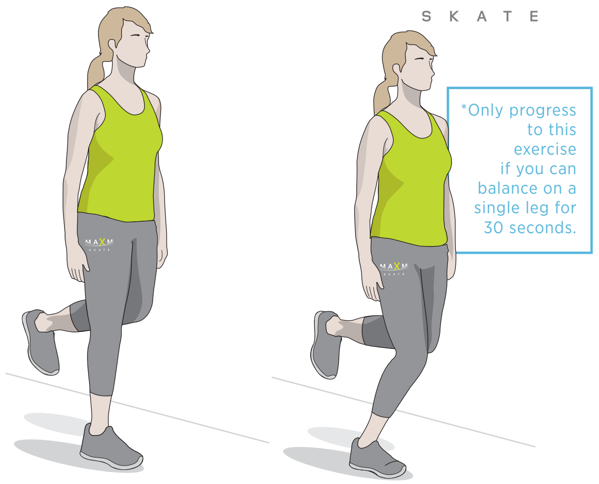

SINGLE LEG KNEE BEND

INSTRUCTION

- Stand on operated leg
- Keep waist/belt line horizontal to the ground & hold for 1 second
- ¼ squat & hold for 1 second
- Return to start position & repeat

FREQUENCY

- 3 sets x 10 reps
- 3 sessions per day

© MAXM Skate Pty Ltd

STRENGTH STAGE 3 - WEEKS 7-12
CALF HIP AND HAMSTRING

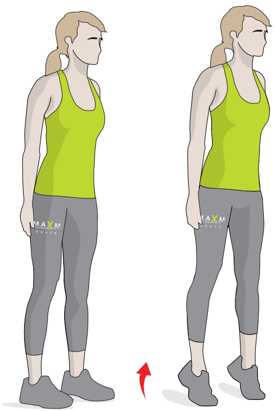

STANDING HEEL RAISE

INSTRUCTION

- Stand with feet shoulder width apart
- Rise slowly onto toes lifting heel as high as possible and hold for 1 second
- Lower slowly to the floor & repeat

FREQUENCY

- 3 sets x 20 reps
- 3 sessions per day

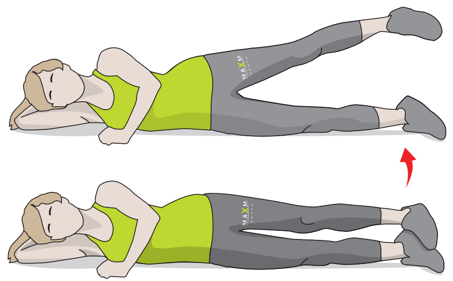

HIP ABDUCTION (SIDE LYING)

INSTRUCTION

- Lay on side with hips perpendicular to the floor
- Bend hips and knees slightly & rotate upper knee upwards turning hip out without moving pelvis
- Lift leg 20 cms & hold for 2 seconds
- Lower slowly, relax & repeat on opposite leg

FREQUENCY

- 3 sets x 10 reps (each leg)
- 3 sessions per day

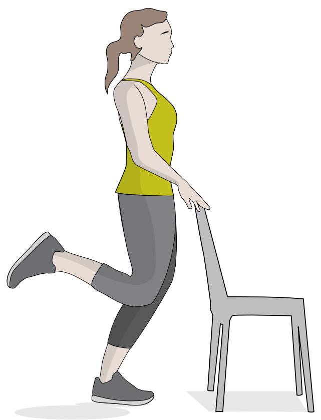

STANDING SUPPORTED HAMSTRING CURL

INSTRUCTION

- Standing in supported position
- Slowly bend operated knee as far as possible
- Hold for 1 second
- Slowly lower to the floor & repeat

FREQUENCY

- 3 sets x 15 reps
- 3 sessions per day

nb: Hamstrings should fatigue

© MAXM Skate Pty Ltd

FUNCTIONAL STAGE 3 - WEEKS 7-12
FUNCTIONAL INTEGRATION

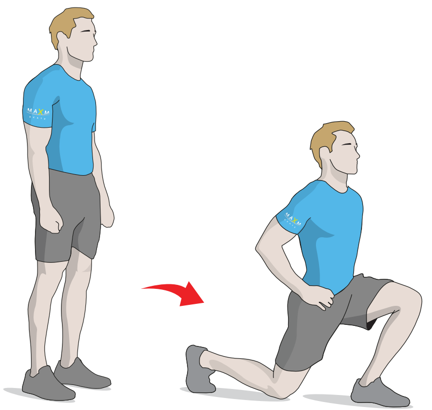

LUNGES

INSTRUCTION

- Stand with feet level
- Slowly step forward on operated leg 50 cms
- Keep waist/ belt line horizontal to the ground
- Bend knee slowly to 30 degrees & hold for 1 second
- Push back to start position. Repeat exercise on non-operated leg

FREQUENCY

- 3 sets x 10 reps (each leg)
- 3 sessions per day

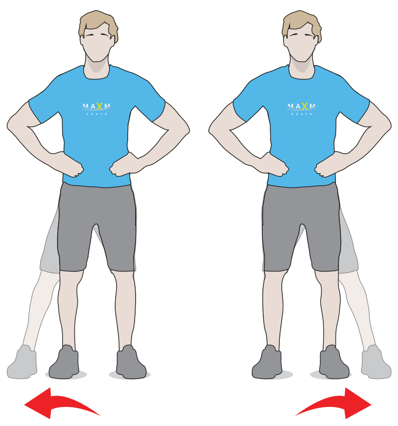

SIDE STEP DRILL

INSTRUCTION

- Stand with feet level using a support
- Slowly step to the right side 30 cm over an imaginary object & return back to start position
- Slowly step to left side 30 cm over an imaginary object & repeat other direction
- Return back to start position & repeat

FREQUENCY

- 3 sets x 10 reps (5 each direction)
- 3 sessions per day

WALKING WITH THE SENSORS ON FOR 15 MINUTES, 3 TIMES PER DAY.

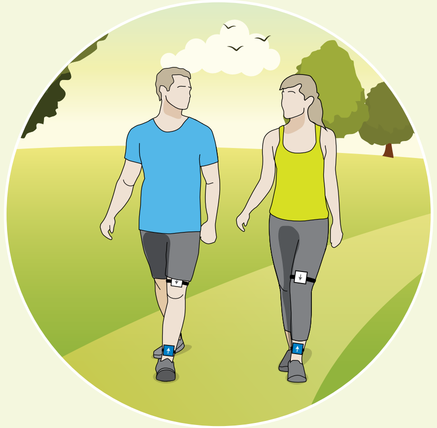

WALKING TARGET VOLUME GOALS
STEPS PER DAY

END WEEK 7	5,000 STEPS PER DAY
END WEEK 8	6,000 STEPS PER DAY
END WEEK 9	7,000 STEPS PER DAY
END WEEK 10	8,000 STEPS PER DAY
END WEEK 11	9,000 STEPS PER DAY
END WEEK 12	10,000 STEPS PER DAY

© MAXM Skate Pty Ltd

M

MAXIMUM

STAGE 4
WEEK 12

© MAXM Skate Pty Ltd

M

MOVEMENT STAGE 4 - WEEKS 12

RANGE OF MOTION AND WARM UP

MAXM SKATE

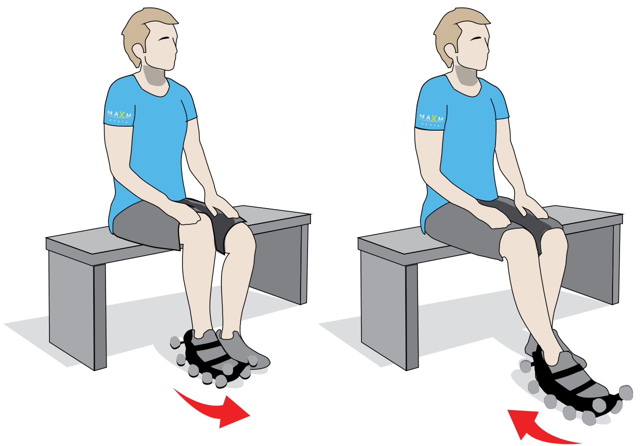

FLAT SKATE

INSTRUCTION

- In a seated position
- Glide skate forward and backward
- Keeping wheels flat on the floor at all times

FREQUENCY

- 3 sets x 40 reps
- Smooth movement, varying speed and range
- 3 sessions per day

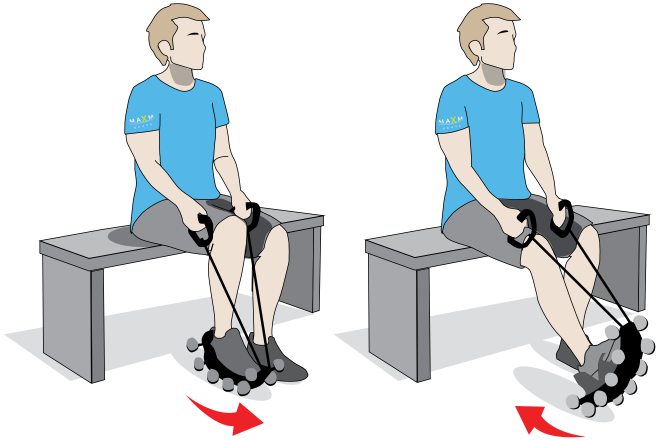

EXTENSION SKATE

INSTRUCTION

- In a seated position place MAXM strap into front hook & glide skate forward
- Engage quadriceps to further straighten knee
- Pull back on the MAXM strap to maximise stretch
- Hold for 2 seconds then release tension
- Return to start position & repeat

FREQUENCY

- 3 sets x 20 reps with 2 secs hold
- 3 sessions per day

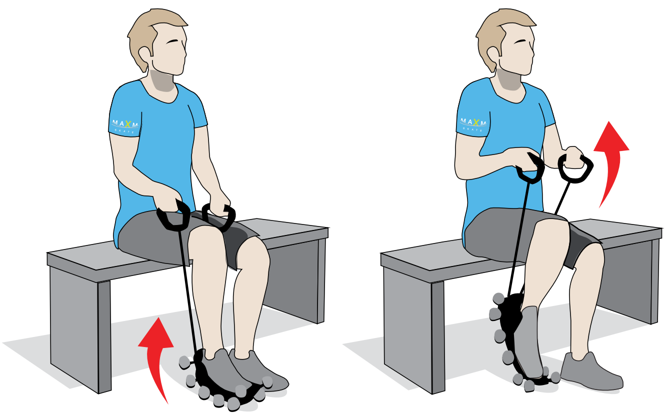

FLEXION SKATE

INSTRUCTION

- In a seated position place MAXM strap into back hook
- Glide skate forward then backwards
- Pull up on MAXM strap to maximise flexion (knee bend)
- Hold for 2 seconds
- Return to start position & repeat

FREQUENCY

- 3 sets x 20 reps with 2 secs hold
- 3 sessions per day

© MAXM Skate Pty Ltd

M

STRENGTH STAGE 4 - WEEKS 12

QUADRICEPS AND SINGLE LEG BALANCE

MAXM SKATE

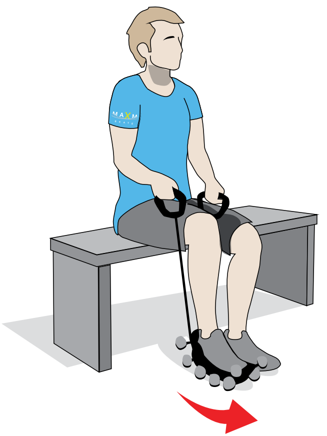

RESISTED EXTENSION SKATE

INSTRUCTION

- Place MAXM strap in back hook & bend knee to 90 degrees
- Provide resistance by pulling on MAXM strap and engage quadricep
- Slowly straighten knee smoothly against resistance as far as possible whilst skate remains flat on floor
- Hold for 2 seconds & maintain tension
- Slowly allowing knee to return to start position

FREQUENCY

- 3 sets x 20 reps
- 3 sessions per day

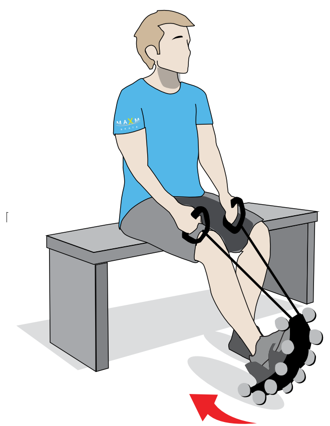

RESISTED FLEXION SKATE

INSTRUCTION

- Place MAXM strap in front hook,
- With no resistance straighten knee
- Pull on MAXM strap & engage hamstring
- Claw/pull heel smoothly against resistance until knee is maximally bent & hold for 2 seconds
- Maintain resistance while slowly allowing skate to return to start position
- Relax & repeat

FREQUENCY

- 3 sets x 20 reps
- 3 sessions per day

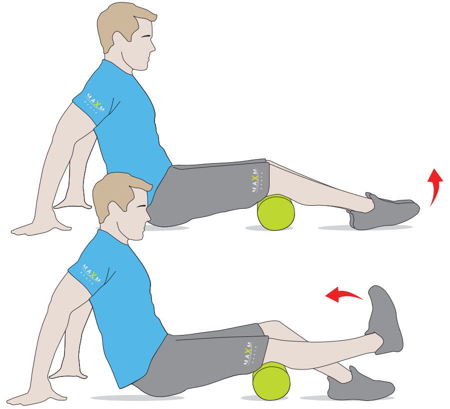

IRQ LONG SITTING

INSTRUCTION

- Pull back toes
- Squeeze inner quad/thigh
- Lift heel slowly, pressing back of thigh into towel
- Hold knee straight 2 seconds
- Lower heel
- Relax & repeat

FREQUENCY

- 3 sets x 20 reps
- 3 sessions per day

© MAXM Skate Pty Ltd

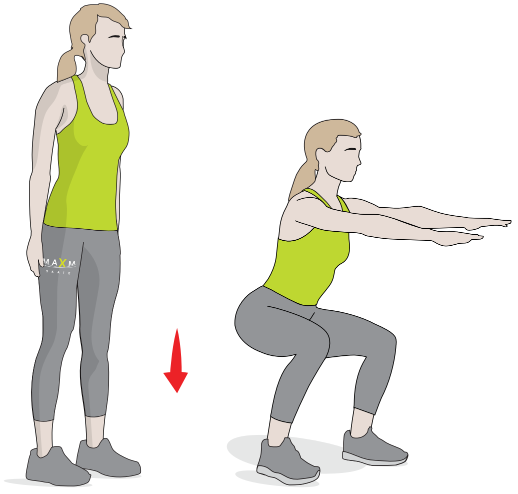

DOUBLE LEG SQUAT

INSTRUCTION

- Stand with feet hip width apart, hands on hips
- Maintain even weight through both heels and bend knees to lower pelvis 30 cms l
- Hold for 2 seconds
- Return to start & repeat

FREQUENCY

- 3 sets x 15 reps
- 3 sessions per day

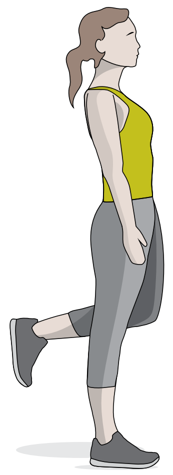

SINGLE LEG BALANCE

INSTRUCTION

- Stand on operated leg
- Balance keeping pelvis level with weight evenly distributed through heel and back of big toe
- Hold for 30 seconds
- Change legs

FREQUENCY

- 3 sets balancing for 30 secs (each leg)
- 3 sessions per day

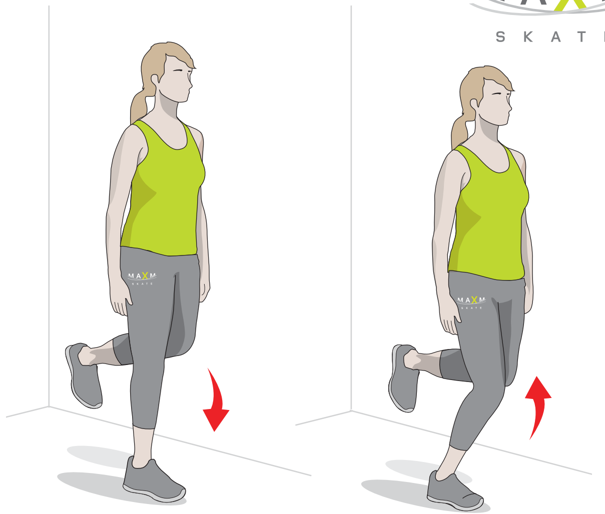

SINGLE LEG BEND

INSTRUCTION

- Stand on operated leg
- Keep waist/belt line horizontal to the ground & hold for 1 second
- ¼ squat & hold for 1 second
- Return to start position & repeat

FREQUENCY

- 3 sets x 15 reps
- 3 sessions per day

© MAXM Skate Pty Ltd

STRENGTH STAGE 4 - WEEKS 12
CALF HIP AND HAMSTRING

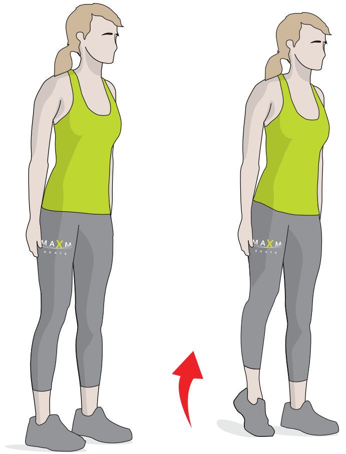

SINGLE HEEL RAISE

INSTRUCTION

- Standing on operated leg facing support
- Slowly lift heel off ground as high as possible pushing through all of big toe
- Hold for 2 seconds
- Lower slowly to the floor & repeat

FREQUENCY

- 3 sets x 10-20 reps
- 3 sessions per day

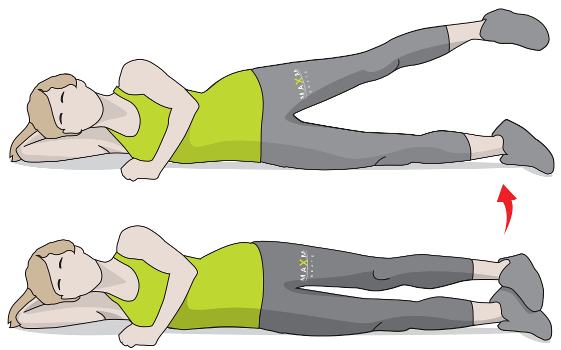

HIP ABDUCTION (SIDE LYING)

INSTRUCTION

- Lay on side with hips perpendicular to the floor
- Bend hips and knees slightly & rotate upper knee upwards turning hip out without moving pelvis
- Lift leg 20 cms & hold for 2 seconds
- Lower slowly, relax & repeat on opposite leg

FREQUENCY

- 3 sets x 10 reps (each leg)
- 3 sessions per day

© MAXM Skate Pty Ltd

FUNCTIONAL STAGE 4 - WEEKS 12
FUNCTIONAL INTEGRATION

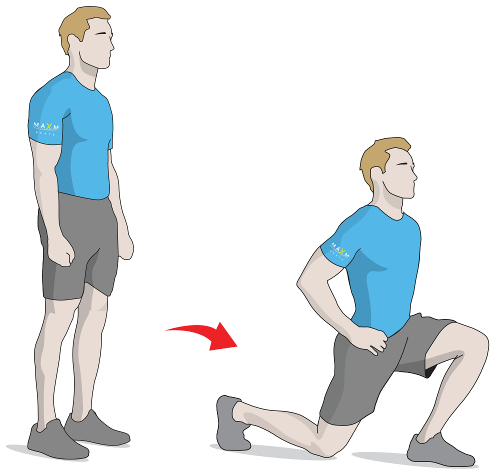

LUNGES

INSTRUCTION

- Stand with feet level
- Slowly step forward on operated leg 50 cms
- Keep waist/ belt line horizontal to the ground
- Bend knee slowly to 30 degrees & hold for 1 second
- Push back to start position. Repeat exercise on non-operated leg

FREQUENCY

- 3 sets x 10 reps (each leg)
- 3 sessions per day

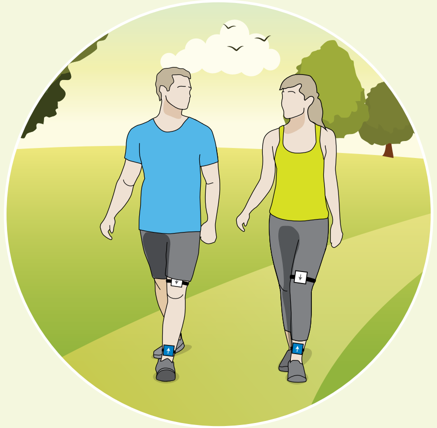

WALKING TARGET VOLUME GOALS
STEPS PER DAY

WEEK 12 ONWARDS
MAINTAIN GREATER THAN
10,000 STEPS PER DAY

© MAXM Skate Pty Ltd

MAXM SKATE

MAXMSKATE.COM.AU

© MAXM Skate Pty Ltd
