# Supplementary material for: The safety, efficacy and cost-effectiveness of the Maxm Skate, a lower limb rehabilitation device for use following total knee arthroplasty: study protocol for a randomised controlled trial
Source: Trials. 2019 Jan 10;20:36. doi: 10.1186/s13063-018-3102-9 (PMC6329189; doi:10.1186/s13063-018-3102-9)
Supplement: Supplementary file 2 — Outpatient Standard Care Physiotherapy Protocol (FPH). (DOCX 15 kb) [file 13063_2018_3102_MOESM2_ESM.docx]

**STANDARD TKR PROTOCOL POST FPH STAY**

**TKR rehabilitation post discharge from FPH varies significantly depending on:**

- Patient progress/safety/ motivation
- Patient preference on rehab approach
- Pain levels
- Previous experiences (i.e. OPPT, rehab)
- Social situation (i.e. residential location, support network, transport options)
- Health insurance

**The aims of continuation of PT are:**

- Exercise progression
- Safety training
- Strength training/ ROM progression
- Manual therapy/ massage
- Hydrotherapy
- Gait retraining
- Proprioception training
- General education

**The main options for continuation of Physiotherapy post FPH discharge are:**

1. Home with Outpatient PT

OPPT Rx includes:

- Usually 1 to 2 session a week for 6 weeks +
- +/- hydrotherapy (1 -2 sessions a week)

1. Home with home visit package

Home visit package includes:

- 2 – 6 weeks of PT visits in the home
- Usually twice a week
- +/- hydrotherapy

1. Inpatient rehabilitation

IP rehab includes:

- 5 days – 3 weeks rehabilitation in a rehab inpatient setting
- Hydrotherapy BD
- Often continuing with Day Rehab post inpatient rehab

1. Day rehabilitation

Day rehab includes:

- 2 to 3 half day rehab sessions a week
- 2 – 6 weeks
- Hydrotherapy

**Regardless of initial Physiotherapy pathway following FPH stay, all patients are encouraged to continue Physiotherapy contact for a minimum of 6 weeks post op.**
